# Protocol for a realist evaluation of a COPD clinical pathway in Saskatchewan, Canada

**DOI:** 10.1371/journal.pone.0359299

**Published:** 2026-10-22

**Authors:** Kathrina Mazurik, Darcy D. Marciniuk, Erika Penz, Patricia Comfort, Alireza Ghorbani-Pashakolaei, Tracey-Ann Stitchell, Tracey Carr, Joshua Shitta, Amy Reid, Gill Westhorp, Collin Hartness, Jason Vanstone, Brenda Andreas, Maggie King, Kim Bell, Johnny Ahale, Ghazale Ahmadalitabar, Stacey McHenry, LUCID Project Team, Gary Groot

**Affiliations:** 1 Department of Community Health and Epidemiology, College of Medicine, University of Saskatchewan, Saskatoon, Saskatchewan, Canada; 2 Division of Respirology and Sleep Medicine, College of Medicine, University of Saskatchewan, Saskatoon, Saskatchewan, Canada; 3 Saskatchewan Health Authority, Regina, Saskatchewan, Canada; 4 Northern Institute, Faculty of Arts and Society, Charles Darwin University, Darwin, Australia; 5 Maple Creek, Saskatchewan, Canada; 6 North Battleford, Saskatchewan, Canada; 7 Regina, Saskatchewan, Canada; University of Colombo Faculty of Medicine, SRI LANKA

## Abstract

Chronic obstructive pulmonary disease (COPD) is a complex and common condition with significant health, social, and economic burdens, especially due to frequent hospitalizations and comorbidities. International research shows current care for COPD is limited by delayed diagnosis, poor coordination among providers, and inconsistent adherence to treatment guidelines, which negatively impacts patient outcomes. Clinical pathways offer a promising solution by improving care coordination, aligning treatment with evidence-based guidelines, and enhancing both patient experiences and system efficiency; however, questions remain about how, for whom, and in which contexts they are most effective. Using a realist evaluation approach, our study will evaluate how, for whom, and in which circumstances an integrated clinical pathway for COPD works in Saskatchewan, Canada. First, we will develop initial program theories based on literature and stakeholder input. Second, we will test these theories using administrative data and realist interviews with healthcare staff, managers, providers, COPD patients, and caregivers. Third, we will refine our theories based on data collected. Fourth, we will translate our findings to enhance future implementation. These phases will be repeated over a three-year span to continuously improve pathway implementation processes and monitor improvements in patient outcomes. Findings will be shared through integrated knowledge translation with system decision-makers and patients. Findings will also be disseminated to the broader research community through conference presentations and peer-reviewed publications.

## Introduction

Chronic obstructive pulmonary disease (COPD) is a progressive respiratory condition marked by persistent airflow limitation, with symptoms such as cough, dyspnea, and sputum production [1,2]. Comorbidities—including cardiovascular disease, diabetes, depression, and anxiety—complicate its course, increasing hospitalizations and care complexity [3,4]. Acute exacerbations are the leading cause of hospitalizations and the main driver of economic burden, with frequent exacerbators representing the highest-cost group [5–7]. The system level burden of COPD is significant, with the Canadian Institute for Health Information estimating $1.5 billion in costs to the healthcare system annually [8].

Studies have identified several factors contributing to suboptimal COPD outcomes. Patients can go undiagnosed (and therefore untreated) for extended periods due to low consideration of COPD by providers, limited access to spirometry testing, lack of confidence and time to interpret spirometry tests, poor quality spirometry testing, or the difficulty of differentiating COPD from other diseases with similar symptoms [9–11]. Attesting to this, a review by Ho. et al. (2019) indicated that rates of under-diagnosis of COPD ranged between 14% and 82% in Canada, with some of this variation attributable to the criterion used [12]. Pharmacological care is often inappropriate or poorly aligned with national and international guidelines [9,10,13]. For instance, Bahremand et al. (2021)’s study in the Canadian province of British Columbia found (through analysis of administrative health databases) that 50% of patients during their first year of diagnosis received prescriptions for inhaled corticosteroids [13], despite Canadian guidelines recommending beginning with longacting muscarinic receptor antagonists (LAMA) or long-acting beta-2 adrenoreceptor agonists (LABA) monotherapy or dual LABA+LAMA therapy if more symptomatic [14]. Fragmented healthcare systems can limit important coordination between specialists, primary care physicians, patients, and community supports [10,15] and providers have called for improved care coordination to access patient action plans, follow-up with patients, and disseminate knowledge about available services [11]. In brief, quality of care could be improved through awareness, care integration, coordination, and guideline adherence.

Improved quality of care also requires support for self-management by patients. Self-management (including adherence to medical regimens, healthy lifestyle changes, action plans, visits with care providers, and exercise) improves patient quality of life and reduces hospitalizations [16]. Despite this, providers often view patient education for self-management as outside of their scope of practice, beyond their expertise, or beyond their skill set [17,18]. Khan et al. reported in a UK-based study that only 40% of COPD patients had self-management plans; 50% of COPD patients had never received advice on diet/exercise; and 43% of current smokers with COPD had been offered practical help to quit smoking in the last year [19]. A study conducted in a Western Canada also found pulmonary rehabilitation to be unequally available to all patients [15]. Managing COPD without such supports is challenging for patients, but even when patients do receive self-management tools or education, their adherence to that guidance varies according to economic position, motivation, information sources, health literacy, and (dis)ability [17,20,21]. Thus, improved patient outcomes require not only broader access to self-management resources, but also tailored supports that reflect psychological and social circumstances [11,21]. Given high variability in the delivery of self-management programs and tools, such as Action Plans [22], and high variability in the self-management profiles of COPD patients [23], evidence-informed guidance for implementing self-management interventions in a targeted fashion could be highly practical for providers and their patients.

Together, patient-level burdens, economic costs, and limitations in quality of care highlight the need for more integrated approaches to COPD care. Clinical pathways offer a multidisciplinary approach to translate evidence-based guidelines into practical interventions [24]. They are designed to enhance quality of care by promoting straightforward, evidence-based practice tailored to the needs of specific populations [25]. Clinical pathways are aimed at stimulating operational efficiency and enhancing equity by reducing variation in care [26]. By encouraging clinical responsibility and establishing accepted standards throughout a patient’s journey, they minimize care fragmentation, optimize cost effectiveness, and improve care throughput [25,27]. This structured approach to care also facilitates patient and family education about the expected course of the condition being managed [28].

Clinical pathways for managing COPD have been widely adopted to improve healthcare outcomes. Studies in Australia, Malaysia, Spain, Belgium, Netherlands, and Singapore have shown that compared to usual care, COPD clinical pathways improve documentation, reduce hospitalization rates, reduce readmission rates, and reduce lengths of stay in hospital [29–33]. According to Ban et al., Malaysian patients in the clinical pathway group had a shorter length of stay than the non-clinical pathway group (median (IQR): 5 (4–7) days versus 7 (7–9) days) and 24.1% less complications (14.7% versus 38.8%) [29]. After 12 months’ follow-up of COPD patients in Spain and Belgium, integrated care showed a lower hospitalisation rate and a higher percentage of patients without re-admissions (49% versus 31%) than usual care without differences in mortality (19% versus 16%, respectively) [30]. In Netherlands, there was a 19.4% reduction in the number of total hospitalization days, without a decrease in health-related quality of life or perceived quality of care [31]. Compared with non-enrolees in Singapore, COPD hospitalisation risk and hospital bed days for integrated care pathway program enrolees was significantly lower in year two [32]. However, Vanhaecht et al. [34] reported that the implementation of an in-hospital care pathway for COPD exacerbation had no impact on the six month readmission rate, but significantly reduced the 30 day readmission rate in Belgium, Italy and Portugal.

In the Canadian province of Saskatchewan, COPD clinical pathways rely on care coordination between primary care providers, respirologists, respiratory therapists, pharmacists, and nurses working collaboratively to reduce healthcare utilization and improve quality of care (see Fig 1) [24]. Recently, Kuwornu et al. evaluated the Saskatchewan COPD pilot pathway’s effects on healthcare use and costs [33,35,36]. They found that, compared to non-pathway patients from the same province, pathway patients had shorter in-patient hospital stays, shorter emergency stays, and fewer drug dispensations [33]. Pathway patients visited doctors and specialists more often than the non-pathway patient group and no differences were found in the *number* of emergency department visits or inpatient hospitalizations for COPD-related reasons between the groups [33]. Kuwornu et al. (in a separate study) found the COPD clinical pathway reduced moderate and severe exacerbations at comparable costs when compared to the *historical* control group; however, compared to *contemporaneous* controls, the pathway achieved marginally better clinical outcomes at slightly higher costs [36]. The researchers additionally found that the pathway reduced COPD-related healthcare costs most for the highest cost patients and that targeted implementation for subgroups (younger patients and patients with comorbidities) would result in the most efficient allocation of resources [33,35]. In summary, studies in Saskatchewan show a mixed but generally positive impact on healthcare utilization, costs, and patient outcomes [33,35,36], in some cases suggesting *for which* patients *in which ways*, and *for what cost* the pathway may be most effective.

**Fig 1:**
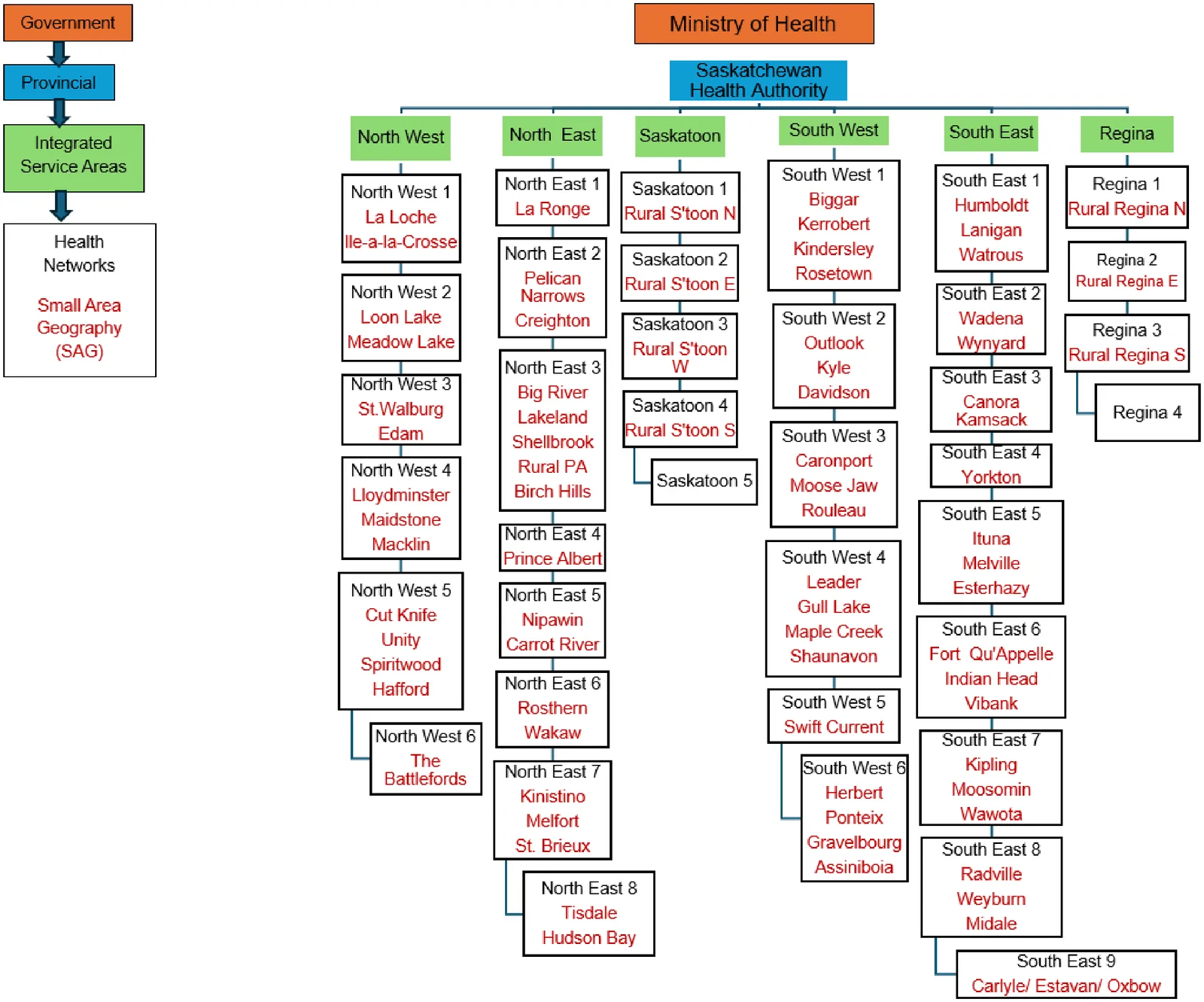
Organization of the healthcare system in Saskatchewan.

Still, there are gaps and limitations in understanding the circumstances driving varied pathway utilization and clinical outcomes. Patient characteristics limiting engagement with chronic disease programs (such as health literacy or economic position) remain unexamined in relation to COPD pathways, limiting interpretation of *for whom* a COPD pathway would be effective [21]. Additionally, implementation processes related to COPD clinical pathways have received little attention, despite their potential to vastly limit intervention effectiveness [37]. Given Saskatchewan’s geographic spread and the 2017 amalgamation of health authorities, the implementation of standardized processes—such as referrals, order sets, testing protocols, reporting standards, and role definitions—is particularly relevant.

To address these gaps in the literature and respond to system-level needs within Saskatchewan’s healthcare context, we will conduct a mixed-method realist evaluation to understand how, why, for whom, and under what circumstances a provincial COPD pathway (and its implementation) works best. By using multiple data collection methods, examining implementation processes, and abstracting program theory from evidence, this study will contribute to academic and applied understandings of clinical pathways. Insights from this study will also inform practices aimed at improving the diagnosis, management, and treatment of COPD, subsequently impacting the quality of care received by COPD patients.

## Methods and analysis

### Realist evaluation approach

Realist evaluation is defined by the aim to investigate not whether an intervention works generally, but rather, “what works, how, in which contexts, and for whom” [38]. Researchers adopting this methodology realize this aim by identifying, testing, and synthesizing program theories – conceptual configurations that relate program mechanisms, contexts, and outcomes. Since these terms are subject to different interpretations [39,40] we offer the following definitions to ensure clarity. We define “mechanisms” as “underlying entities, processes, or structures which operate in particular contexts to generate outcomes of interest” [41]. We view “mechanisms” as constituted by combinations of resources provided by a program and participants’ reasoning and responses to those resources [39]. Following Pawson and Tilley, we define “outcomes” as the intended and unintended consequences of programs that arise when mechanisms are activated within particular contexts [42]. Finally, we define “contexts” as “the relational and dynamic features that dynamically shap[e] the mechanisms through which [an] intervention works” [40]. From this standpoint, contexts refer not merely to the setting of an intervention, but rather *what it is about* the setting that shapes the way mechanisms trigger outcomes, where the context is most likely to be an evolving – rather than stable or controllable – condition. Taken together, these conceptualizations of contexts (as dynamic), mechanisms (as mutually constituted by people and the resources available to them), and outcomes (as intended, unintended, positive, and negative) affirm a view of programs as complex, open systems.

### Study setting

This study will be conducted in the province of Saskatchewan, Canada. Saskatchewan is one of 10 provinces in Canada with a population of 1,253,569 [43] with a single-payer government funded and delivered healthcare system for all residents except for members of the Canadian Armed Forces, Royal Canadian Mounted Police, First Nations’ peoples, and residents of the federal justice system. In 2017, Saskatchewan’s 12 health authorities were consolidated into a single provincial health authority to reduce administration and improve service coordination [44,45]. The province is organized into 38 regional networks, which exist to support team-based care tailored to community needs [46].

### Program description

The Saskatchewan COPD Clinical Pathway was launched in April 2025 as part of a Provincial Clinical Network, with local health networks implementing it in a staggered fashion following the launch. The pathway was developed to standardize prevention, assessment, diagnosis, and management across care settings. It includes education, commercial tobacco cessation, vaccination, spirometry testing, pulmonary rehabilitation, and self-management supports. Patients may enter via self-referral, primary care, emergency, urgent care, acute care, or HealthLine 811, with referrals routed through local access teams, who connect patients with local services and ensure timely follow-up. Interdisciplinary chronic disease management teams (some of which are embedded in primary care sites) deliver education, testing, and ongoing care to referred patients. These teams may include respiratory educators, respiratory therapists, exercise therapists, nurses, nurse practitioners, physiotherapists, occupational therapists, paramedics, licensed practical nurses, critical care associates, and administrative staff, with roles aligning to local capacity and referral needs. The pathway emphasizes early identification with trusted spirometry, coordinated and seamless transitions between acute and community care, hospital discharge bundles, self-management tools, and flexible supports, such as virtual follow-up and home health monitoring [47]. It also integrates culturally specific resources for commercial tobacco cessation and palliative care guidance for advanced disease. In line with research suggesting that patient and family engagement can enhance care and access to care [48] and that involvement of clinical and management staff facilitates program and technology implementation [49–51], the COPD pathway was developed in collaboration with patient and family partners, community-based organizations (e.g., LungSask, Pharmacy Association of Saskatchewan, Saskatchewan Cancer Agency, Saskatchewan Medical Association), research groups (e.g., University of Saskatchewan Division of Respirology and Sleep Medicine, and Canadian Centre for Rural and Agricultural Health), and other stakeholders (e.g., Home Oxygen companies, Healthline 811, Rx Files, the Ministry of Health).

### Patient and public involvement

This research is being led by an Implementation Science Team made up of academic researchers, healthcare providers, health system administrators, and patient family partners. During the grant-writing stage, stakeholders from each of these four groups contributed to the global research objectives and methods. Each will also be consulted to inform program theory development, advise on appropriate recruitment and data collection techniques, and contribute to knowledge translation. A steering group made up of members of each of the four groups will gather regularly to monitor global project progress and suggest mitigation strategies as obstacles arise.

### Overview of study phases

This study has been planned in accordance with the RAMESES reporting standards by Wong et al. (2016) [52]. The evaluation will begin with a scoping phase to develop a rough program theory. Stakeholder consultation and literature reviews will identify hypothesized contexts, mechanisms, and outcomes and their inter-relations. Following this scoping phase, the evaluation will proceed through iterative learning cycles of data collection, analysis, and knowledge translation. Each cycle will use mixed methods, involving 1) administrative data (to track outcome trends) and 2) qualitative interviews (to explore the contexts and mechanisms that explain those patterns). Analysis will involve refining context-mechanism-outcome (CMO) configurations to bring the program theory into alignment with evidence. In each cycle, findings will be shared with stakeholders to inform adjustments to the implementation of the COPD pathway as well as to spur new evaluation questions. Fig 2 presents these phases.

**Fig 2:**
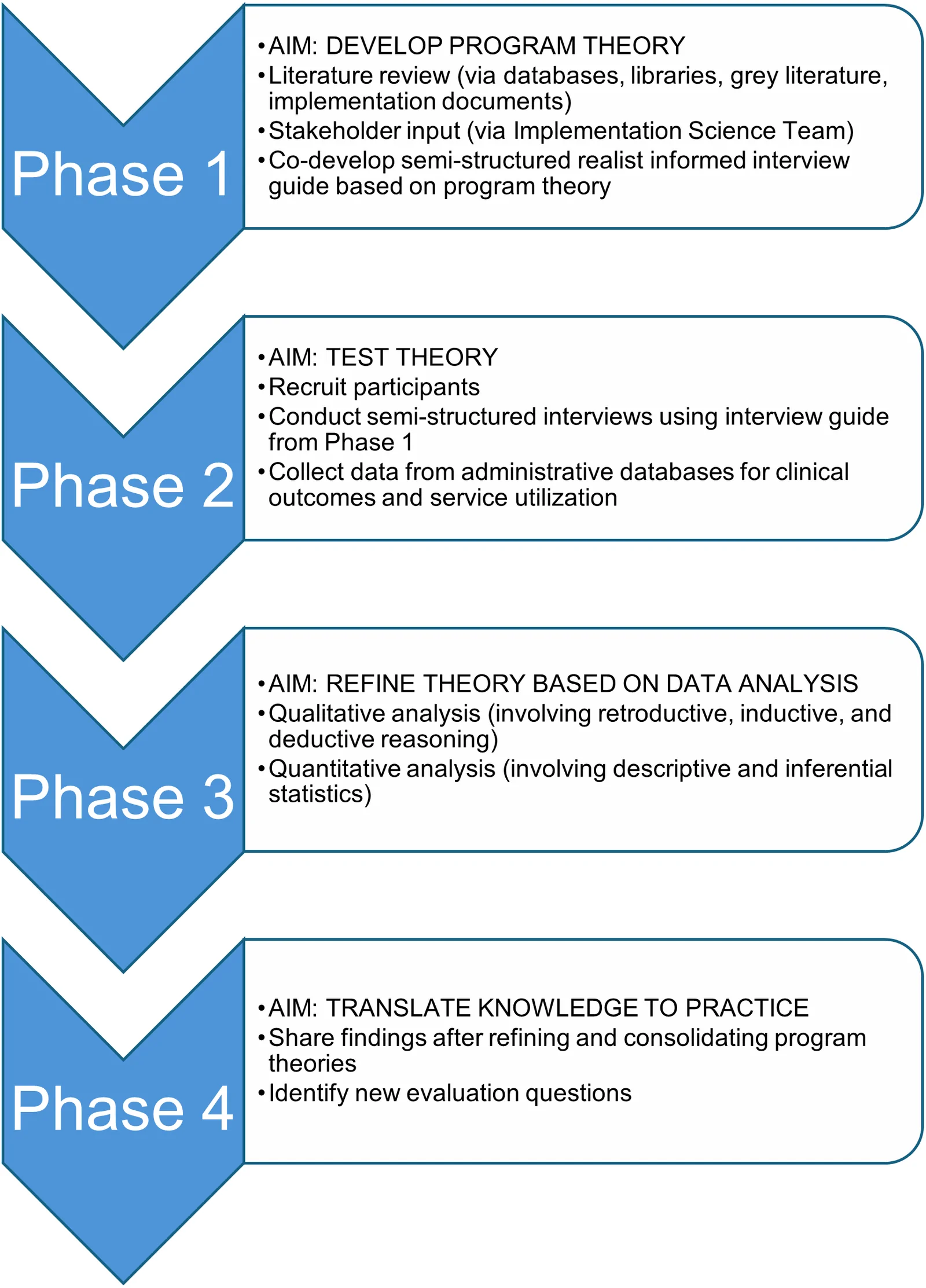
Summary of study phases within each learning cycle.

At the time of submission, recruitment and data collection were occurring for the first learning cycle. Recruitment of participants for the first learning cycle began December 1^st^ 2025 and will end by June 30^th^ 2026. Findings from the first learning cycle will be fully disseminated by September 30^th^ 2026. A new learning cycle will begin October 1^st^ 2026. Each phase within the learning cycle is detailed in Table 1.

**Table 1:** Complete study timeline.

Phase	Timeline																
	2025				2026				2027				2028				2029
	Q1	Q2	Q3	Q4	Q1	Q2	Q3	Q4	Q1	Q2	Q3	Q4	Q1	Q2	Q3	Q4	Q1
1																	
2					**												
3																	
4																	
	LEARNING CYCLE							LEARNING CYCLE				LEARNING CYCLE					

** Current status.

### Phase 1: Program theory development

#### Literature review.

In this stage, the objective is to gather information from existing literature and stakeholder consultation to develop causal propositions that will later be tested. In the first learning cycle, searches will be guided by five questions inspired by the RE-AIM framework, interpreted here as a taxonomy of outcomes relevant to health interventions, where those outcomes are Reach, Effectiveness, Adoption, Implementation, and Maintenance [53]. The questions are as follows:

1. How, why, and in what circumstances do healthcare providers and organizations adopt or fail to adopt COPD clinical pathways into practice?
2. How, why, and in what circumstances do healthcare organizations successfully implement COPD clinical pathways as intended (e.g., with respect to consistency of delivery, time, cost of implementation)?
3. How, why, for whom, and in what circumstances do COPD clinical pathways reach or fail to reach COPD patients?
4. How, why, for whom, and in what circumstances do COPD clinical pathways improve patients’ clinical outcomes, patient-reported experiences, and patient-reported outcomes?
5. How, why, for whom, and in what circumstances is the effectiveness of COPD clinical pathways sustained over time at both the individual (patient, healthcare providers) and organizational levels?

In following learning cycles, searches will be guided by questions that have been prioritized by the Implementation Science Team and steering group during knowledge translation (see Phase 4). For the literature reviews in all learning cycles, data extraction templates will be followed to collect general information (i.e., objective; data collection and analysis methods; settings; participants; theoretical frameworks; evidence of richness, rigor, and relevance) and evidence identifying Contexts, Outcomes, and Mechanisms. Causal pathway diagrams and explanatory statements will be developed to represent the program theories [54,55]. Subsequent targeted searches will examine specific parts of the program theory with greater precision.

#### Consultations with stakeholders.

Consultations with stakeholders within the Implementation Science Team will be used to gather feedback on preliminary CMO configurations derived from literature searches. Stakeholders will be invited to identify areas requiring clarification and suggest additional directions for targeted searching.

### Phase 2: Data Collection to Test Theory

To test the initial program theories, interview data and administrative data will be collected (see Table 2 for summary).

**Table 2:** Evidence-gathering techniques.

Phase	Source of theory	Contexts	Mechanisms	Outcomes
1: Initial program theory development	Literature review (informed by stakeholder consultation)	✓	✓	✓
2: Program theory testing	Interviews with COPD patients and caregivers	✓	✓	✓
	Interviews with COPD pathway implementers and adopters	✓	✓	✓
	Administrative healthcare data			✓

#### Realist interviews.

Realist interviews will be used to test the contexts and causal mechanisms contributing to outcomes [56]. The sample of participants will be chosen to represent specific contexts that surface in the initial program theory development phase. We will aim to recruit a minimum of one participant and a maximum of ten participants to represent each context of interest. This range accords with a realist’s need to optimize explanatory power (i.e., where even a single “case” can provide a rich and relevant account of real causal processes) while being constrained by project resources [57]. Should the initial program theory suggest an extraneous number of contexts of relevance, the steering group (in consultation with the rest of the Implementation Science Team) will prioritize contexts such that no more than 50 interviews are conducted in each learning cycle (with a maximum 150 interviews for the entire duration of the study to adhere to the project budget). The selection of interview participants will be continually revisited in each learning cycle according to new questions and theories that arise, permitting the researchers to resolve gaps and contradictions between evidence and theory [57]. Recruitment channels will include community advertising, local chronic disease management teams, personal outreach by the Implementation Science Team, and the Provincial Clinical Network for Chronic Disease (a group of over 150 healthcare providers improving chronic disease care in the province, with COPD as the initial focus).

Prior to each interview, a study coordinator will explain the nature of the interview process and ask a series of screening questions to determine whether the participant fits into a context relevant to theory-testing. If the participant fits a context under investigation, they will be invited to participate in an interview over the phone or Zoom. A consent form will be shared with the participant prior to the interview time. Participants will have the opportunity to provide written consent by signing and sharing the form back with the researcher prior to the interview. If the participant does not provide written consent prior to the scheduled interview time, the researcher will review the consent form with the participant at the beginning of the interview time and, after addressing any questions or concerns, the researcher will ask for the participant’s consent and document it by signing and dating the consent form as a witness. Whether the consent is obtained verbally or in written form, the participant will receive a copy of the signed consent form for their records. The interview will begin by broadly identifying participants’ personal and work-related interactions with the pathway [56]. After this, the interviewer will pose questions to identify relevant contexts, mechanisms, and outcomes evidenced by the interviewees’ stories and examples [56].

#### Administrative data.

Administrative data will be used to capture implementation and clinical effectiveness outcomes from September 2017 to August 2028. This date aligns with the launch of a COPD care pathway pilot in Regina (the second largest city in the province) and therefore takes account of variability the pilot pathway would have introduced. Healthcare administrative databases will include hospital discharge abstracts, ambulatory care records, physician billing claims, outpatient prescription drug dispensation records, and population registration files. These data sets cover all Saskatchewan residents regardless of whether their care is paid for by the provincial or federal government. Data anticipated to be relevant to the study include: records of COPD diagnoses, records of comorbidities, hospital readmissions, hospital length of stay, emergency department visits, physician visits, specialist visits, spirometry tests, pulmonary function tests, and region. Data on COPD patient referrals and other implementation processes will be provided by the Saskatchewan Health Authority. Data will be anonymously linked between the datasets through a unique, masked health number. Informed consent has been waived by the Saskatchewan Health Authority for the use of anonymized healthcare administrative data.

### Phase 3: Data analysis and theory refinement

#### Administrative data analysis.

Administrative health data will be analyzed using longitudinal statistical methods to examine COPD outcomes over time. Descriptive analyses will summarize participants and cohort characteristics using means and standard deviations, with correlation analysis used to identify patterns between outcomes and settings with different contextual features. Regression models may also be used given sufficient data and alignment with realist thinking that 1) assumes the possibility of non-linear relationships between phenomena, 2) cautions against interpreting causality from association, and 3) attends to heterogeneity more than average effects [58].

#### Qualitative data analysis.

Qualitative data collected in phase 2 will be stored, transcribed, and analyzed in NVivo v. 12 and Excel spreadsheets. Inductive, deductive, and retroductive reasoning will be applied to identify key contextual factors and underlying mechanisms that generate observed outcomes [59–61]. An initial open coding stage will be conducted to organise the data and facilitate retrieval of relevant excerpts for later analysis (essentially, the data will be indexed). During this stage, data will be labelled using terms derived from transcripts rather than being deductively assigned to existing program theories or their components. Provisional coding will first be developed by a single researcher using a subset of transcripts, after which other team members will apply and refine the coding framework across the remaining data through iterative discussions and revisions. The second stage will apply a deductive approach to test and refine initial program theories developed in the Phase 1. Each researcher will focus on one pre-existing CMO configuration, systematically retrieving relevant excerpts and organizing them in evidence tables, where the excerpt will be analysed to abstract context, mechanism, and outcome elements, including partial configurations (C-M and M-O combinations or contextual descriptions that lend details to other causal linkages) where complete CMO configurations are not evident. Where gaps exist, transcripts will be revisited and retroductive reasoning will be used and explicitly acknowledged to infer missing elements. Individual CMO configurations will then be consolidated and the consolidated CMO configuration will be presented to other researchers and relevant stakeholders (alongside relevant and/or illustrative data) for feedback and questions to drive further rounds of revision. To address data unrelated to the initial program theories, researchers will formulate and test “hunches” that relate at least two parts of a CMO [62], using the same evidence tables and process described prior. The same process for consolidating, presenting, and soliciting feedback will occur.

### Phase 4: Knowledge translation

This phase will involve consolidating findings and presenting refined program theories and supporting evidence to COPD pathway leads and patient family partners who are part of the Implementation Science Team. These individuals will use the evidence to inform real-time adjustments to the COPD clinical pathway. The research team will work closely with them to identify which outputs are most useful and actionable for decision-making and implementation. During this phase, new questions will be identified to initiate the following learning cycle.

## Conclusion

COPD clinical pathways offer significant potential to improve patient outcomes, prevent unnecessary hospitalizations, support primary care practitioners, enhance patient outcomes, and optimize resource use; however, mixed findings regarding their effectiveness suggest underlying complexities that merit more investigation. Notably, further evaluation is needed to understand 1) *which* patient groups benefit most from these pathways (e.g., high-need, high-complexity patients vs. patients requiring more preventative care; poorly-resourced settings vs. well-resourced health settings), 2) *which* implementation strategies facilitate adoption and implementation by *which* providers (and why), and 3) *how* pathways implemented at a larger scale may depend on the presence of unique conditions in comparison to single-site pathway implementations (for example, requiring more investment in relationship-building or requiring more standard operating procedures). By linking context, mechanisms, and outcomes, we will help create more effective, equitable, and scalable integrated care pathways, both in Saskatchewan and beyond.

We anticipate several unique strengths for this study. First, whereas prior research has primarily examined the outcomes and costs of COPD clinical pathways, this approach will link contexts, mechanisms, and outcomes through causal theory. Second, implementation processes and outcomes will be examined in addition to the evaluation of the clinical pathway itself, extending the current scope of knowledge. Third, both quantitative and qualitative data will be drawn on to capture explanatory processes and monitor success in implementation and outcomes. Fourth, insights will be delivered to the healthcare system in a rapid, responsive manner, enabling adjustments to both the pathway and the implementation strategies supporting it. We also anticipate limitations to the study. Notably, given the potentially vast study scope, the final program theories may have to reflect targeted priorities for the health system and evaluation team, as opposed to reflecting a comprehensive account of causal processes at all levels of conceptual abstraction. The reader should note that any amendments to the study protocol will be documented and reported to the ethics board and other relevant committees. These changes will also be stated in subsequent protocols or reports.

## References

[1] Barstow C, Forbes D. Respiratory conditions: chronic obstructive pulmonary disease. FP Essent. 2019;486:26–32. 31710455

[2] Jo YS. Long-term outcome of chronic obstructive pulmonary disease: a review. Tuberc Respir Dis (Seoul). 2022;85(4):289–301. doi: 10.4046/trd.2022.0074 35822318 PMC9537656

[3] Hillas G, Perlikos F, Tsiligianni I, Tzanakis N. Managing comorbidities in COPD. Int J Chron Obstruct Pulmon Dis. 2015;10:95–109. doi: 10.2147/COPD.S54473 25609943 PMC4293292

[4] Yawn B. Strategies to improve outcomes in COPD. J Fam Pract. 2022;71. 10.12788/jfp.0413 35960950

[5] Tran DT, Thanh NX, Ohinmaa A, Mayers I, Jacobs P. Current and future direct healthcare cost burden of chronic obstructive pulmonary disease in Alberta, Canada. Can J Respir Crit Care Sleep Med. 2019;4(1):39–47. doi: 10.1080/24745332.2019.1629850

[6] May SM, Li JTC. Burden of chronic obstructive pulmonary disease: healthcare costs and beyond. Allergy Asthma Proc. 2015;36(1):4–10. doi: 10.2500/aap.2015.36.3812 25562549 PMC5554331

[7] Iheanacho I, Zhang S, King D, Rizzo M, Ismaila AS. Economic Burden of Chronic Obstructive Pulmonary Disease (COPD): A systematic literature review. Int J Chron Obstruct Pulmon Dis. 2020;15:439–60. doi: 10.2147/COPD.S234942 32161455 PMC7049777

[8] Canadian Institute for Health Information. COPD Focus High Users - Infographic. 2017. https://www.cihi.ca/en/copd-a-focus-on-high-users-infographic

[9] Meiwald A, Gara-Adams R, Rowlandson A, Ma Y, Watz H, Ichinose M. Qualitative validation of COPD evidenced care pathways in Japan, Canada, England, and Germany: common barriers to optimal COPD care. Int J Chron Obstruct Pulmon Dis. 2022;17:1507–21. doi: 10.2147/COPD.S360983 PMC9255283 35801119

[10] Shahaj O, Meiwald A, Puri Sudhir K, Gara-Adams R, Wark P, Cazaux A, et al. Mapping the common barriers to optimal COPD care in high and middle-income countries: qualitative perspectives from clinicians. Int J Chron Obstruct Pulmon Dis. 2024;19:1207–23. doi: 10.2147/COPD.S449659 38831892 PMC11146611

[11] Vachon B, Giasson G, Gaboury I, Gaid D, Noël De Tilly V, Houle L, et al. Challenges and Strategies for Improving COPD primary care services in Quebec: Results of the experience of the COMPAS+ quality improvement collaborative. Int J Chron Obstruct Pulmon Dis. 2022;17:259–72. doi: 10.2147/COPD.S341905 35140460 PMC8819163

[12] Ho T, Cusack RP, Chaudhary N, Satia I, Kurmi OP. Under- and over-diagnosis of COPD: a global perspective. Breathe (Sheff). 2019;15(1):24–35. doi: 10.1183/20734735.0346-2018 30838057 PMC6395975

[13] Bahremand T, Etminan M, Roshan-Moniri N, De Vera MA, Tavakoli H, Sadatsafavi M. Are COPD prescription patterns aligned with guidelines? Evidence from a Canadian population-based study. Int J Chron Obstruct Pulmon Dis. 2021;16:751–9. doi: 10.2147/COPD.S290805 PMC8006812 33790551

[14] Bourbeau J, Bhutani M, Hernandez P, Aaron SD, Beauchesne M-F, Kermelly SB, et al. 2023 Canadian Thoracic Society Guideline on Pharmacotherapy in Patients With Stable COPD. Chest. 2023;164(5):1159–83. doi: 10.1016/j.chest.2023.08.014 37690008

[15] Hartford W, Asgarova S, MacDonald G, Berger M, Cristancho S, Nimmon L. Macro and meso level influences on distributed integrated COPD care delivery: a social network perspective. BMC Health Serv Res. 2021;21(1):491. doi: 10.1186/s12913-021-06532-y 34024272 PMC8141100

[16] Sánchez-Nieto JM, Andújar-Espinosa R, Bernabeu-Mora R, Hu C, Gálvez-Martínez B, Carrillo-Alcaraz A, et al. Efficacy of a self-management plan in exacerbations for patients with advanced COPD. Int J Chron Obstruct Pulmon Dis. 2016;11:1939–47. doi: 10.2147/COPD.S104728 27574418 PMC4994798

[17] Ogunbayo OJ, Russell S, Newham JJ, Heslop-Marshall K, Netts P, Hanratty B, et al. Understanding the factors affecting self-management of COPD from the perspectives of healthcare practitioners: a qualitative study. NPJ Prim Care Respir Med. 2017;27(1):54. doi: 10.1038/s41533-017-0054-6 28924245 PMC5603550

[18] Russell S, Ogunbayo OJ, Newham JJ, Heslop-Marshall K, Netts P, Hanratty B, et al. Qualitative systematic review of barriers and facilitators to self-management of chronic obstructive pulmonary disease: views of patients and healthcare professionals. NPJ Prim Care Respir Med. 2018;28(1):2. doi: 10.1038/s41533-017-0069-z 29343739 PMC5772437

[19] Khan A, Dickens AP, Adab P, Jordan RE. Self-management behaviour and support among primary care COPD patients: cross-sectional analysis of data from the Birmingham Chronic Obstructive Pulmonary Disease Cohort. NPJ Prim Care Respir Med. 2017;27(1):46. doi: 10.1038/s41533-017-0046-6 28729620 PMC5519687

[20] Chen X, Wen N, Liu J, He Y, Wei L, Zhang R, et al. Self-management and COPD: a qualitative study to explore the perceived barriers and recommendations to improve COPD management using the Information-Motivation-Behavioral skills model. NPJ Prim Care Respir Med. 2025;35(1):34. doi: 10.1038/s41533-025-00443-9 40715100 PMC12297598

[21] Goodridge D, Bandara T, Marciniuk D, Hutchinson S, Crossman L, Kachur B, et al. Promoting chronic disease management in persons with complex social needs: A qualitative descriptive study. Chron Respir Dis. 2019;16. doi: 10.1177/1479973119832025 30836794 PMC6402059

[22] Yusup I, Tang R, Kouri A, Sin DD, Marciniuk DD, Gupta S. COPD Action plans: gaps in development methods, content, and format. Int J Chron Obstruct Pulmon Dis. 2026;21:571223. doi: 10.2147/COPD.S571223 41878302 PMC13006551

[23] Yu J, Fu J, Zhao S, Zhang S, Li Q, Zhang D, et al. Latent classes of self-management and associated factors in patients with COPD. BMC Pulm Med. 2025;26(1):34. doi: 10.1186/s12890-025-04074-1 41466390 PMC12859867

[24] Rotter T, Plishka C, Hansia MR, Goodridge D, Penz E, Kinsman L, et al. The development, implementation and evaluation of clinical pathways for chronic obstructive pulmonary disease (COPD) in Saskatchewan: protocol for an interrupted times series evaluation. BMC Health Serv Res. 2017;17(1):782. doi: 10.1186/s12913-017-2750-x 29183318 PMC5704544

[25] Rotter T, Jong RB de, Lacko SE, Ronellenfitsch U, Kinsman L. Clinical pathways as a quality strategy. Improv Healthc Qual Eur Charact Eff Implement Differ Strateg. 2019. https://www.ncbi.nlm.nih.gov/books/NBK549262/ Accessed 2024 December 18.

[26] Lawal AK, Rotter T, Kinsman L, Machotta A, Ronellenfitsch U, Scott SD, et al. What is a clinical pathway? Refinement of an operational definition to identify clinical pathway studies for a Cochrane systematic review. BMC Med. 2016;14:35. doi: 10.1186/s12916-016-0580-z 26904977 PMC4765053

[27] Rotter T, Kinsman L, James E, Machotta A, Gothe H, Willis J, et al. Clinical pathways: effects on professional practice, patient outcomes, length of stay and hospital costs. Cochrane Database Syst Rev. 2010;(3):CD006632. doi: 10.1002/14651858.CD006632.pub2 20238347

[28] Dobesh PP, Bosso J, Wortman S, Dager WE, Karpiuk EL, Ma Q, et al. Critical pathways: the role of pharmacy today and tomorrow. Pharmacotherapy. 2006;26(9):1358–68. doi: 10.1592/phco.26.9.1358 16945060

[29] Ban A, Ismail A, Harun R, Abdul Rahman A, Sulung S, Syed Mohamed A. Impact of clinical pathway on clinical outcomes in the management of COPD exacerbation. BMC Pulm Med. 2012;12:27. doi: 10.1186/1471-2466-12-27 22726610 PMC3479064

[30] Casas A, Troosters T, Garcia-Aymerich J, Roca J, Hernández C, Alonso A, et al. Integrated care prevents hospitalisations for exacerbations in COPD patients. Eur Respir J. 2006;28(1):123–30. doi: 10.1183/09031936.06.00063205 16611656

[31] Westbroek LF, Klijnsma M, Salomé P, Sekhuis LM, Rolink E, Korsmit E, et al. Reducing the number of hospitalization days for COPD: setting up a transmural-care pathway. Int J Chron Obstruct Pulmon Dis. 2020;15:2367–77. doi: 10.2147/COPD.S242914 33061354 PMC7533231

[32] Wu CX, Hwang CH, Tan WS, Tai KP, Kwek LSL, Chee TG, et al. Effectiveness of a chronic obstructive pulmonary disease integrated care pathway in a regional health system: a propensity score matched cohort study. BMJ Open. 2018;8(3):e019425. doi: 10.1136/bmjopen-2017-019425 29572394 PMC5875646

[33] Kuwornu JP, Maldonado F, Cooper EJ, Groot G, Penz E, Reid A, et al. Impacts of chronic obstructive pulmonary disease care pathway on healthcare utilization and costs: a matched multiple control cohort study in Saskatchewan, Canada. Value Health. 2023;26(9):1334–44. doi: 10.1016/j.jval.2023.04.013 37187234

[34] Vanhaecht K, Lodewijckx C, Sermeus W, Decramer M, Deneckere S, Leigheb F, et al. Impact of a care pathway for COPD on adherence to guidelines and hospital readmission: a cluster randomized trial. Int J Chron Obstruct Pulmon Dis. 2016;11:2897–908. doi: 10.2147/COPD.S119849 27920516 PMC5126002

[35] Kuwornu JP, Maldonado F, Groot G, Cooper EJ, Penz E, Sommer L, et al. An economic evaluation of chronic obstructive pulmonary disease clinical pathway in Saskatchewan, Canada: Data-driven techniques to identify cost-effectiveness among patient subgroups. PLoS One. 2024;19(4):e0301334. doi: 10.1371/journal.pone.0301334 38557914 PMC10984414

[36] Kuwornu JP, Maldonado F, Groot G, Penz E, Cooper EJ, Reid A. Real-world cost-consequence analysis of an integrated chronic disease management program in Saskatchewan, Canada. Health Serv Insights. 2024;17. doi: 10.1177/11786329231224621 PMC10785729 38223214

[37] Morton K, MacNeill S, Sanderson E, Dixon P, King A, Jenkins S, et al. Evaluation of “care bundles” for patients with chronic obstructive pulmonary disease (COPD): a multisite study in the UK. BMJ Open Respir Res. 2019;6(1):e000425. doi: 10.1136/bmjresp-2019-000425 31258918 PMC6561386

[38] Pawson R. Invisible mechanisms. Eval J Australas. 2008;8:1–13.

[39] Dalkin SM, Greenhalgh J, Jones D, Cunningham B, Lhussier M. What’s in a mechanism? Development of a key concept in realist evaluation. Implement Sci. 2015. doi: 10.1186/s13012-015-0237-x PMC4408605 25885787

[40] Greenhalgh J, Manzano A. Understanding ‘context’ in realist evaluation and synthesis. Int J Soc Res Methodol. 2022;25:583–95. doi: 10.1080/13645579.2021.1918484

[41] Astbury B, Leeuw FL. Unpacking black boxes: mechanisms and theory building in evaluation. Am J Eval. 2010;31:363–81. doi: 10.1177/1098214010371972

[42] Pawson R, Tilley N. Realistic evaluation. SAGE. 1997.

[43] Saskatchewan Population. Gov. Sask. 2025. https://dashboard.saskatchewan.ca/people-community/people/population Accessed 2025 July 14.

[44] Government announces move to single provincial health authority. Gov. Sask. 2017. https://www.saskatchewan.ca/government/news-and-media/2017/january/04/single-health-authority

[45] Patients won’t yet see changes wtih single Sask. health authority, CEO says. CBC News. 2017. https://www.cbc.ca/news/canada/saskatchewan/new-single-health-authority-board-1.4432002 Accessed 2025 July 14.

[46] Health Networks in Saskatchewan. 2021. https://www.saskhealthauthority.ca/your-health/conditions-illnesses-services-wellness/all-z/health-networks-team-based-care/health-networks-saskatchewan

[47] Sask. Health Auth. Chronic Obstructive Pulmonary Disease (COPD) Pathway. https://www.saskhealthauthority.ca/your-health/conditions-illnesses-services-wellness/chronic-obstructive-pulmonary-disease-copd-pathway

[48] Bombard Y, Baker GR, Orlando E, Fancott C, Bhatia P, Casalino S, et al. Engaging patients to improve quality of care: a systematic review. Implement Sci. 2018;13(1):98. doi: 10.1186/s13012-018-0784-z 30045735 PMC6060529

[49] Evans-Lacko S, Jarrett M, McCrone P, Thornicroft G. Facilitators and barriers to implementing clinical care pathways. BMC Health Serv Res. 2010;10:182. doi: 10.1186/1472-6963-10-182 20584273 PMC2912894

[50] Laukka E, Huhtakangas M, Heponiemi T, Kanste O. Identifying the roles of healthcare leaders in HIT implementation: a scoping review of the quantitative and qualitative evidence. Int J Environ Res Public Health. 2020;17(8):2865. doi: 10.3390/ijerph17082865 32326300 PMC7215383

[51] Lu AD, Kaul B, Reichert J, Kilbourne AM, Sarmiento KF, Whooley MA. Implementation strategies for frontline healthcare professionals: people, process mapping, and problem solving. J Gen Intern Med. 2021;36(2):506–10. doi: 10.1007/s11606-020-06169-3 32918200 PMC7878661

[52] Wong G, Westhorp G, Manzano A, Greenhalgh J, Jagosh J, Greenhalgh T. RAMESES II reporting standards for realist evaluations. BMC Med. 2016;14(1):96. doi: 10.1186/s12916-016-0643-1 27342217 PMC4920991

[53] Nillsen P, Augustsson H. Implementation strategies and outcomes. In: Nillsen, P, editor. Implementation science: Theory and application. London: Routledge; 2024. pp. 99–113. doi: 10.4324/9781003318125

[54] Punton M, Isabel V, Michaelis C, Boydell E. Reality bites: Making realist evaluation useful in the real world. CDI Pract Pap. 2020;1–13.

[55] Meza RD, Klasnja P, Lewis CC, Pullman MD, Mettert KD, Hawkes R, et al. Causal pathway diagrams to understand how implementation strategies work. In: Nillsen, P, editor. Implementation science: Theory and application. London: Routledge; 2024. pp. 114–24. doi: 10.4324/9781003318125

[56] Manzano A. The craft of interviewing in realist evaluation. Evaluation. 2016;22(3):342–60. doi: 10.1177/1356389016638615

[57] Emmel N. Sampling and choosing cases in qualitative research: a realist approach. SAGE. 2013.

[58] Westhorp G, Feeny S. Using surveys in realist evaluation. Eval J Australas. 2025;25:45–64. doi: 10.1177/1035719X241292083

[59] Dhakal K. NVivo. J Med Libr Assoc. 2022; 110:270–2. doi: 10.5195/jmla.2022.1271 PMC9014916 35440911

[60] Examining the Application of Retroductive Theorizing in Realist-Informed Studies. http://ouci.dntb.gov.ua/en/works/ldNEDBm7/ Accessed 2025 January 14.

[61] Rycroft-Malone J, Seers K, Eldh AC, Cox K, Crichton N, Harvey G, et al. A realist process evaluation within the Facilitating Implementation of Research Evidence (FIRE) cluster randomised controlled international trial: an exemplar. Implement Sci. 2018;13(1):138. doi: 10.1186/s13012-018-0811-0 30442165 PMC6238283

[62] Dalkin S, Forster N, Hodgson P, Lhussier M, Carr SM. Using computer assisted qualitative data analysis software (CAQDAS; NVivo) to assist in the complex process of realist theory generation, refinement and testing. Int J Soc Res Methodol. 2021;24:123–34. doi: 10.1080/13645579.2020.1803528

