# Supplementary material for: Protocol for a realist evaluation of a COPD clinical pathway in Saskatchewan, Canada
Source: PLoS One. 2026 Oct 22;21(10):e0359299. doi: 10.1371/journal.pone.0359299 (PMC13690240; doi:10.1371/journal.pone.0359299)
Supplement: Supplementary file 2 [file pone.0359299.s002.docx]

**September 10^th^ 2026**

**Below are our responses to the editor:**

Please check these

1. In the tracked-changes manuscript, Candace Skrapek is included in the author list (Line 18), but she is omitted from both the clean manuscript author list and the "Order of Authors" metadata on Editorial Manager. Clarify whether she meets ICMJE criteria for authorship or if her contribution belongs solely under Acknowledgements (where she is currently thanked in Line 449).

**Skrapek has been removed as an author and is listed under Acknowledgments instead. This is reflected in the tracked changes document – Sept 10 version as well as the clean copy.**

2. Affiliation numbers in the tracked-changes manuscript for Maggie King (5), Kim Bell (5), Brenda Andreas (5), and Candace Skrapek (5) clash with the numbered institutional keys (where key 5 is listed as Maple Creek, 6 as North Battleford, and 7 as Regina). Ensure numeric superscript affiliations match the corresponding city/institution entries across all versions.

**The affiliation numbers align (Andreas=5; King=6, Bell=7) and this is reflected in the tracked changes document – Sept 10 version and the clean copy.**

3. In the clean manuscript text, Figure 1 (Line 193) and Figure 2 (Line 249) are embedded via placeholder text, Hope you will be providing original figures separately.

**We confirm that the figures have been uploaded separately.**

4. Table 2 is formatted twice in the tracked-changes version (once in a clear block and once scattered across lines 345–351 due to line-break artefacts during typesetting). Ensure the stray text from the duplicate table in the revision draft is completely removed.

**This appears to be a non-issue in the clean version and when “no mark-up” setting is activated in the tracked-changes version.**

5. Line 46: "Findings will be shared through integrated knowledge transition..." — The correct, standard implementation science terminology is Integrated Knowledge Translation (IKT) rather than "knowledge transition."

**We have made this correction.**

6. Line 207: "...emphasizes early identification with available spirometry with trusted spirometry..." — Contains accidental repetitive wording. Rephrase to "emphasizes early identification with available, trusted spirometry..."

**We have made this change.**

7. Line 310: "...a minimum of one participant and a maximum of ten participants to represent each context of interest." — Typo in double spacing between "of" and "ten".

**This has been resolved.**

8. To strengthen the body of the manuscript (Lines 345–377), consider adding a brief statement on data/theoretical saturation within realist sampling logic (i.e., recruitment within each context continues until conceptual depth and theory-testing capacity are achieved).

**The concept of “theoretical saturation” draws from grounded theory methodology. The key texts we’ve drawn on to inform our global approach and approach to sampling make no mention of this concept and we prefer our current phrasing, which emphasizes the resolution of gaps and contradictions between evidence and theory. See Manzano (2016), Mukumbang et al. (2016), and Stoszkowski & Collins (2022) as examples of realist researchers who have, similarly, eschewed the concept of “saturation” in favor of criteria or processes that fit more cleanly within the realist paradigm.**

9. In the Methods section (Lines 330–338), specify the exact date range for historical baseline data (e.g., September 2017 to present) to clarify how pre-pathway baseline outcomes will be compared against post-implementation trends in Saskatchewan.

**We have revised “present” to “August 2028”.**

10. Verify that the RAMESES II checklist is uploaded as a Supporting Information file (S1 File) and explicitly referenced in the manuscript text under Methods, as required by PLOS ONE for realist study designs.

**We can confirm the RAMESES reporting standards are uploaded as a supplementary file, however there is no “checklist” as these are published in rubric form. The RAMESES reporting standards are referenced in the Overview of study phases within the section Methods and analysis.**

[Note: HTML markup is below. Please do not edit.]

Reviewers' comments:

Reviewer's Responses to Questions

**Comments to the Author**

1. Does the manuscript provide a valid rationale for the proposed study, with clearly identified and justified research questions?

The research question outlined is expected to address a valid academic problem or topic and contribute to the base of knowledge in the field.

Reviewer #1: Yes

2. Is the protocol technically sound and planned in a manner that will lead to a meaningful outcome and allow testing the stated hypotheses?

The manuscript should describe the methods in sufficient detail to prevent undisclosed flexibility in the experimental procedure or analysis pipeline, including sufficient outcome-neutral conditions (e.g. necessary controls, absence of floor or ceiling effects) to test the proposed hypotheses and a statistical power analysis where applicable. As there may be aspects of the methodology and analysis which can only be refined once the work is undertaken, authors should outline potential assumptions and explicitly describe what aspects of the proposed analyses, if any, are exploratory.

Reviewer #1: Yes

3. Is the methodology feasible and described in sufficient detail to allow the work to be replicable?


Descriptions of methods and materials in the protocol should be reported in sufficient detail for another researcher to reproduce all experiments and analyses. The protocol should describe the appropriate controls, sample size calculations, and replication needed to ensure that the data are robust and reproducible.

Reviewer #1: Yes

4. Have the authors described where all data underlying the findings will be made available when the study is complete?

The [PLOS Data policy](https://can01.safelinks.protection.outlook.com/?url=https%3A%2F%2Ftrack.editorialmanager.com%2FCL0%2Fhttps%3A%252F%252Fjournals.plos.org%252Fplosone%252Fs%252Fmaterials-and-software-sharing%2F1%2F010f01a065ec24a6-cc2dcffc-6977-4786-a7a8-d057b8a71063-000000%2F5gT5oC-ZFwFnpxpjtZHPsCBo8FZwmnkPQVq1iyr_8D4%3D258&data=05%7C02%7Ckathrina.mazurik%40usask.ca%7C63218ba8e8254c08339c08df09835064%7C24ab6cd0487e47229bc3da9c4232776c%7C0%7C0%7C639240131964492957%7CUnknown%7CTWFpbGZsb3d8eyJFbXB0eU1hcGkiOnRydWUsIlYiOiIwLjAuMDAwMCIsIlAiOiJXaW4zMiIsIkFOIjoiTWFpbCIsIldUIjoyfQ%3D%3D%7C0%7C%7C%7C&sdata=Qx2dnQ9aKil%2Bld%2BDLZMZNDycS3PPMCYnrXjHjZYjnzA%3D&reserved=0) requires authors to make all data underlying the findings described in their manuscript fully available without restriction, with rare exception, at the time of publication. The data should be provided as part of the manuscript or its supporting information, or deposited to a public repository. For example, in addition to summary statistics, the data points behind means, medians and variance measures should be available. If there are restrictions on publicly sharing data—e.g. participant privacy or use of data from a third party—those must be specified.

Reviewer #1: Yes

5. Is the manuscript presented in an intelligible fashion and written in standard English?

PLOS ONE does not copyedit accepted manuscripts, so the language in submitted articles must be clear, correct, and unambiguous. Any typographical or grammatical errors should be corrected at revision, so please note any specific errors here.

Reviewer #1: Yes

6. Review Comments to the Author

Please use the space provided to explain your answers to the questions above and, if applicable, provide comments about issues authors must address before this protocol can be accepted for publication. You may also include additional comments for the author, including concerns about research or publication ethics.

You may also provide optional suggestions and comments to authors that they might find helpful in planning their study.


(Please upload your review as an attachment if it exceeds 20,000 characters)

Reviewer #1: I want to appreciate the attention to detail and the effort from the authors to answer the review and address the comments and suggestions made.

Regarding the question about “Descriptions of methods and materials in the protocol should be reported in sufficient detail for another researcher to reproduce all experiments and analyses”, it was more related to the reproducibility of methods. I understand the relevance and importance of publishing this study protocol and I appreciate the effort to improve the transparency of the methodology, particularly the data analysis section.

I just recommend a last review of text and spacing throughout the manuscript (noticed a space missing in line 73).

I consider this a relevant study protocol, and it should be published. I’m eager to see its results. I wish the authors success in this project.

We thank the reviewer for the comments.
We have added a space on line 73 between “symptomatic” and the in-text citation.
