# Supplementary material for: Protocol for a realist evaluation of a COPD clinical pathway in Saskatchewan, Canada
Source: PLoS One. 2026 Oct 22;21(10):e0359299. doi: 10.1371/journal.pone.0359299 (PMC13690240; doi:10.1371/journal.pone.0359299)
Supplement: Supplementary file 3 [file pone.0359299.s001.docx]

June 11^th^ 2026

We thank the editor and reviewer for their review of the paper and notes for improvement. We have addressed the feedback below, with our responses highlighted in yellow and references to the changes in the manuscript (line numbers) included in parenthesis.

We have also appended a change to authorship form that outlines our requests for change and their justifications.

Kathrina Mazurik

**EDITOR FEEDBACK**

1. Please ensure that your manuscript meets PLOS ONE's style requirements, including those for file naming. The PLOS ONE style templates can be found at

[https://journals.plos.org/plosone/s/file?id=wjVg/PLOSOne_formatting_sample_main_body.pdf](https://can01.safelinks.protection.outlook.com/?url=https%3A%2F%2Ftrack.editorialmanager.com%2FCL0%2Fhttps%3A%252F%252Fjournals.plos.org%252Fplosone%252Fs%252Ffile%253Fid%3DwjVg%252FPLOSOne_formatting_sample_main_body.pdf%2F1%2F010f019daf73089c-f202b2d4-48ad-4eee-ab85-4a6b2041ee29-000000%2F4P4VSStpUxVfYZ308LLM7Yr2aR8Wz1hpFilFxSIWHuY%3D258&data=05%7C02%7Ckathrina.mazurik%40usask.ca%7Cbdfc1baad07a4ebca08908de9f8b75a3%7C24ab6cd0487e47229bc3da9c4232776c%7C0%7C0%7C639123618827276032%7CUnknown%7CTWFpbGZsb3d8eyJFbXB0eU1hcGkiOnRydWUsIlYiOiIwLjAuMDAwMCIsIlAiOiJXaW4zMiIsIkFOIjoiTWFpbCIsIldUIjoyfQ%3D%3D%7C0%7C%7C%7C&sdata=r3rFkGt0KWu028CFexK9spuKslfqEPXkaAMVXUPEOLo%3D&reserved=0) and [https://journals.plos.org/plosone/s/file?id=ba62/PLOSOne_formatting_sample_title_authors_affiliations.pdf](https://can01.safelinks.protection.outlook.com/?url=https%3A%2F%2Ftrack.editorialmanager.com%2FCL0%2Fhttps%3A%252F%252Fjournals.plos.org%252Fplosone%252Fs%252Ffile%253Fid%3Dba62%252FPLOSOne_formatting_sample_title_authors_affiliations.pdf%2F1%2F010f019daf73089c-f202b2d4-48ad-4eee-ab85-4a6b2041ee29-000000%2FeX2AmyO37Jd-0mvoh1hR18cug-WJRGochhNn01Jmw0w%3D258&data=05%7C02%7Ckathrina.mazurik%40usask.ca%7Cbdfc1baad07a4ebca08908de9f8b75a3%7C24ab6cd0487e47229bc3da9c4232776c%7C0%7C0%7C639123618827298273%7CUnknown%7CTWFpbGZsb3d8eyJFbXB0eU1hcGkiOnRydWUsIlYiOiIwLjAuMDAwMCIsIlAiOiJXaW4zMiIsIkFOIjoiTWFpbCIsIldUIjoyfQ%3D%3D%7C0%7C%7C%7C&sdata=r4AU76GAgaSH88BBaKshg5BwW7pFvyNFpoMXj2uz%2B20%3D&reserved=0)

We have aligned our formatting with the above guidelines (see tracked changes throughout document).

2. Thank you for stating the following financial disclosure:

[This research has been funded by Canadian Institutes of Health Research grant#IT7 - 188084.].

Please state what role the funders took in the study.  If the funders had no role, please state: "The funders had no role in study design, data collection and analysis, decision to publish, or preparation of the manuscript."

If this statement is not correct you must amend it as needed.

Please include this amended Role of Funder statement in your cover letter; we will change the online submission form on your behalf.

Please revise the Role of Funder statement to the following : “This research has been funded by Canadian Institutes of Health Research grant #IT7 -188084. The funders had no role in study design, data collection and analysis, decision to publish, or preparation of the manuscript.”

3. Please remove any funding-related text from the manuscript. Funding information should not appear in any section or other areas of your manuscript. We will only publish funding information present in the Funding Statement section of the online submission form.

We have removed this information.

4. Thank you for stating the following in the Competing Interests section:

[I have read the journal's policy and the authors of this manuscript have the following competing interests: Authors comprise a mix of dual‑role program developer/implementer–evaluators and individuals focused exclusively on evaluation. This distribution of roles mitigates potential conflicts of interest.].

Please confirm that this does not alter your adherence to all PLOS ONE policies on sharing data and materials, by including the following statement: "This does not alter our adherence to PLOS ONE policies on sharing data and materials.” (as detailed online in our guide for authors <http://journals.plos.org/plosone/s/competing-interests>).  If there are restrictions on sharing of data and/or materials, please state these. Please note that we cannot proceed with consideration of your article until this information has been declared.

Please include your updated Competing Interests statement in your cover letter; we will change the online submission form on your behalf.

Please revise the Competing Interests statement to the following: “I have read the journal's policy and the authors of this manuscript have the following competing interests: Authors comprise a mix of dual‑role program developer/implementer–evaluators and individuals focused exclusively on evaluation. This distribution of roles mitigates potential conflicts of interest. This does not alter our adherence to PLOS ONE policies on sharing data and materials.”

5. Please amend your list of authors on the manuscript to ensure that each author is linked to an affiliation. Authors’ affiliations should reflect the institution where the work was done (if authors moved subsequently, you can also list the new affiliation stating “current affiliation:….” as necessary).

The Patient Partners on our team do not have a formal affiliation to any institution (e.g., University of Saskatchewan or Saskatchewan Health Authority). Therefore, we have revised the list of authors to link these patient partners to their towns/cities of residence to highlight geographic representation (lines #14-17).

6. Your ethics statement should only appear in the Methods section of your manuscript. If your ethics statement is written in any section besides the Methods, please move it to the Methods section and delete it from any other section. Please ensure that your ethics statement is included in your manuscript, as the ethics statement entered into the online submission form will not be published alongside your manuscript.

We have removed the additional, unnecessary Ethics & Dissemination section and transferred to the Methods section all content that was not already captured in Methods (i.e., line #324).

7. We note you have included a table to which you do not refer in the text of your manuscript. Please ensure that you refer to Tables 1-2 in your text; if accepted, production will need this reference to link the reader to the Tables.

We have added references to the tables in the body of the manuscript (lines #247 and #288).

8. Please include captions for your Supporting Information files at the end of your manuscript, and update any in-text citations to match accordingly. Please see our Supporting Information guidelines for more information: <http://journals.plos.org/plosone/s/supporting-information>.

Our figures (2) and tables (2) are intended to be included in the body of the script. We have no supporting information files to share.

**REVIEWER FEEDBACK**

[Note: HTML markup is below. Please do not edit.]

Reviewers' comments:

Reviewer's Responses to Questions

**Comments to the Author**

1. Does the manuscript provide a valid rationale for the proposed study, with clearly identified and justified research questions?

The research question outlined is expected to address a valid academic problem or topic and contribute to the base of knowledge in the field.

Reviewer #1: Yes

2. Is the protocol technically sound and planned in a manner that will lead to a meaningful outcome and allow testing the stated hypotheses?

The manuscript should describe the methods in sufficient detail to prevent undisclosed flexibility in the experimental procedure or analysis pipeline, including sufficient outcome-neutral conditions (e.g. necessary controls, absence of floor or ceiling effects) to test the proposed hypotheses and a statistical power analysis where applicable. As there may be aspects of the methodology and analysis which can only be refined once the work is undertaken, authors should outline potential assumptions and explicitly describe what aspects of the proposed analyses, if any, are exploratory.

Reviewer #1: Yes

3. Is the methodology feasible and described in sufficient detail to allow the work to be replicable?


Descriptions of methods and materials in the protocol should be reported in sufficient detail for another researcher to reproduce all experiments and analyses. The protocol should describe the appropriate controls, sample size calculations, and replication needed to ensure that the data are robust and reproducible.

Reviewer #1: No

We will address this note with both 1) a response regarding our purpose for publishing a protocol and 2) some revisions that (though unrequested by the reviewer) support these objectives more fully.

The purpose of publishing this protocol is not to impose a rigid or positivist structure on the study, but rather to promote transparency, methodological clarity, and accountability to community partners, participants, and readers. In the context of partnership-based and patient-oriented research, transparently sharing protocols helps ensure relational accountability by clearly documenting the study’s commitments to collaboration, organization-to-organization respect, and patient engagement from the outset. While realist research is inherently iterative and evolves throughout the research process, this protocol outlines the guiding methodological framework and engagement-related commitments of the study, while recognizing that method will respond to the emerging theoretical insights throughout the analytic process. In addition, we anticipated the final research manuscript may exceed the word limits of many journals. Therefore, referring to the protocol serves a practical purpose to ensure that our methods are described in detail and available to all readers.

We have taken into consideration whether there is an opportunity to improve our transparency of analysis. As a result, we have added detail to our plan for statistical analysis, including realist assumptions that must be attended to while conducting this analysis, which otherwise might be violated (lines #345-352). In addition, we have added details to the analysis of qualitative data and the steps through which inductive, deductive, and retroductive reasoning will be applied (lines #355-377).

4. Have the authors described where all data underlying the findings will be made available when the study is complete?

The [PLOS Data policy](https://can01.safelinks.protection.outlook.com/?url=https%3A%2F%2Ftrack.editorialmanager.com%2FCL0%2Fhttps%3A%252F%252Fjournals.plos.org%252Fplosone%252Fs%252Fmaterials-and-software-sharing%2F1%2F010f019daf73089c-f202b2d4-48ad-4eee-ab85-4a6b2041ee29-000000%2FDzxAQwo87936kssnnRilRTICPOn_sib6Lt90IFqlJmY%3D258&data=05%7C02%7Ckathrina.mazurik%40usask.ca%7Cbdfc1baad07a4ebca08908de9f8b75a3%7C24ab6cd0487e47229bc3da9c4232776c%7C0%7C0%7C639123618827459855%7CUnknown%7CTWFpbGZsb3d8eyJFbXB0eU1hcGkiOnRydWUsIlYiOiIwLjAuMDAwMCIsIlAiOiJXaW4zMiIsIkFOIjoiTWFpbCIsIldUIjoyfQ%3D%3D%7C0%7C%7C%7C&sdata=MpdrrBE3Mdca5Y4uzaH%2FHqIFwCYuU1bp%2BkpVA%2BcaDes%3D&reserved=0) requires authors to make all data underlying the findings described in their manuscript fully available without restriction, with rare exception, at the time of publication. The data should be provided as part of the manuscript or its supporting information, or deposited to a public repository. For example, in addition to summary statistics, the data points behind means, medians and variance measures should be available. If there are restrictions on publicly sharing data—e.g. participant privacy or use of data from a third party—those must be specified.

Reviewer #1: Yes

5. Is the manuscript presented in an intelligible fashion and written in standard English?

PLOS ONE does not copyedit accepted manuscripts, so the language in submitted articles must be clear, correct, and unambiguous. Any typographical or grammatical errors should be corrected at revision, so please note any specific errors here.

Reviewer #1: Yes

6. Review Comments to the Author

Please use the space provided to explain your answers to the questions above and, if applicable, provide comments about issues authors must address before this protocol can be accepted for publication. You may also include additional comments for the author, including concerns about research or publication ethics.

You may also provide optional suggestions and comments to authors that they might find helpful in planning their study.

(Please upload your review as an attachment if it exceeds 20,000 characters)

Reviewer #1: Congratulations on the article and on the research you propose to carry out. Integrated healthcare in COPD is crucial to improving, for example, adherence and the long-term effects of interventions such as pulmonary rehabilitation and self-management programs. It will be very interesting to understand how you plan to implement these clinical pathways in this setting and what impact they might have locally. I believe this article is well-written, scientifically relevant, and should be published, with some revisions:

Introduction
- Line 59-60 - Could you present some data on estimates of undiagnosed patients?

We have added a Canada-relevant range drawn from a review article (lines #64-65).

- Line 63 - How is pharmacological care inappropriate or poorly aligned? Some practical examples could be useful.
We have added some specific findings from a relevant Canadian study on this topic (lines #67-73).

- Line 80 - It could be interesting to mention the lack of clear guidelines on how to implement these self-management interventions.

We have added a comment to this effect and cited two relevant studies that speak to the variability of self-management interventions and the variability of patients’ self-management profiles (lines #91-95).

- Line 126-128 - Were stakeholders involved in the development of this clinical pathway? It would be a good opportunity to frame, with evidence, the importance of developing health interventions and solutions that involve stakeholders, in order to improve adherence and effectiveness.

We have indicated the various collaborations that were part of developing the pathway and also highlighted the value of this stakeholder engagement by drawing on two review studies (lines #214-219).

Methods:
- Are there any reporting guidelines you could use to guide the study’s development and reporting?

We have added a statement at the outset of the Methods regarding our adherence to RAMESES reporting standards (lines #231-232).

- Line 192-194 - How is the coordination made between different health isntituitions? Was there an improvement in digital services, for example, was interoperability accounted for?

We have clarified that the improvements in coordination are largely due to increased capacity and trusted interpretation with centralized referrals for spirometry, spirometry test results being readily available over electronic health record viewers, enhanced transition between acute and community care, and hospital discharge bundles (lines #207-211).

- Line 229 - What does data collection consist of at this stage? Interviews? Who are the stakeholders included? Yous should present this information in a more comprehensive manner.
In Phase 1, there is no data collection as in realist evaluation the purpose is to initially form rough program theories. We have added a global statement to reinforce this (lines #255-256), used clearer language in Table 2 (so that it is consistent with what is in the text), clarified that the stakeholders who are part of the consultation are within the Implementation Science Team (line #282) and moved the table earlier in the text so that it more clearly sign-posts the following write-up.

- Line 266-267 - Is there an evidence-based justification for this sample size decision?

We have provided a justification that aligns with realist understandings of sampling (lines #298-301).

Conclusion
- The first paragraph could describe more comprehensively and clearly the clinical and future research implications of this study.

We have added further details to highlight the potential research implications of this study as well noting that these pathways also may serve primary care practitioners (lines #388-398).

Line 348-349 - Are there any barriers to implementing the protocol? Is there a mitigation plan for those risks?

We do not foresee any barriers implementing the protocol.

Typos:
- Line 90 "throughput"

This was intentional spelling, meant to characterize the number of patients that can be treated within the system (as opposed to “throughout” – as in care “throughout” the system).

- Line 162 - "shap[e] the mechanisms through which [an]"

We have included the square brackets to indicate that the quotation has been adapted grammatically.

- Line 224 - "leraning"

We have corrected the error.

7. PLOS authors have the option to publish the peer review history of their article ([what does this mean?](https://can01.safelinks.protection.outlook.com/?url=https%3A%2F%2Ftrack.editorialmanager.com%2FCL0%2Fhttps%3A%252F%252Fjournals.plos.org%252Fplosone%252Fs%252Feditorial-and-peer-review-process%2523loc-peer-review-history%2F1%2F010f019daf73089c-f202b2d4-48ad-4eee-ab85-4a6b2041ee29-000000%2FqvsYYjBZJqDdWcSaY_IxDnQMR32_e3zMo_DLHDIRpBw%3D258&data=05%7C02%7Ckathrina.mazurik%40usask.ca%7Cbdfc1baad07a4ebca08908de9f8b75a3%7C24ab6cd0487e47229bc3da9c4232776c%7C0%7C0%7C639123618827483188%7CUnknown%7CTWFpbGZsb3d8eyJFbXB0eU1hcGkiOnRydWUsIlYiOiIwLjAuMDAwMCIsIlAiOiJXaW4zMiIsIkFOIjoiTWFpbCIsIldUIjoyfQ%3D%3D%7C0%7C%7C%7C&sdata=DkgnJnZuHZ4SPW0OiWn3inhkJl3bS6a11wwQ0eUkkrg%3D&reserved=0)). If published, this will include your full peer review and any attached files.

If you choose “no”, your identity will remain anonymous but your review may still be made public.

**Do you want your identity to be public for this peer review?** For information about this choice, including consent withdrawal, please see our [Privacy Policy](https://can01.safelinks.protection.outlook.com/?url=https%3A%2F%2Ftrack.editorialmanager.com%2FCL0%2Fhttps%3A%252F%252Fwww.plos.org%252Fprivacy-policy%2F1%2F010f019daf73089c-f202b2d4-48ad-4eee-ab85-4a6b2041ee29-000000%2FYBwrLNtFymI-M2r2REIAKbEyHOKEegDI24mqCgK4Kfc%3D258&data=05%7C02%7Ckathrina.mazurik%40usask.ca%7Cbdfc1baad07a4ebca08908de9f8b75a3%7C24ab6cd0487e47229bc3da9c4232776c%7C0%7C0%7C639123618827507896%7CUnknown%7CTWFpbGZsb3d8eyJFbXB0eU1hcGkiOnRydWUsIlYiOiIwLjAuMDAwMCIsIlAiOiJXaW4zMiIsIkFOIjoiTWFpbCIsIldUIjoyfQ%3D%3D%7C0%7C%7C%7C&sdata=THdJJD5dUwrGBrU1CQ%2B5SFG6HC6oKNLC9UbdhlYcfho%3D&reserved=0).

Reviewer #1: No

**Additional minor changes:**

In addition to the above, we have included minor revisions:

- We clarified that the 43% of patients who didn’t receive smoking cessation support was *among current smokers* with COPD (as opposed to the denominator reflecting the entire group, including non-smokers) (line #88)
- We added precision to our wording concerning the relationship between evidence and theory (line #162)
- We clarified that program theories will also be presented to patient family partners who are part of the implementation science team (not only pathway leads)
- We corrected an address for one of the authors and a middle initial for one of the authors
- We corrected a duplicate citation of Ban et al. (2012) (formerly reference # 25 and #32)
