# Supplementary material for: Protocol for a realist evaluation of a COPD clinical pathway in Saskatchewan, Canada
Source: PLoS One. 2026 Oct 22;21(10):e0359299. doi: 10.1371/journal.pone.0359299 (PMC13690240; doi:10.1371/journal.pone.0359299)
Supplement: Peer review history

# Peer review: Decision Letter 0

**DOI:** 10.1371/journal.pone.0359299.r001

**Authors:** Jennifer Tucker

21 Apr 2026

Dear Dr. Mazurik,

Thank you for submitting your manuscript to PLOS ONE. After careful consideration, we feel that it has merit but does not fully meet PLOS ONE’s publication criteria as it currently stands. Therefore, we invite you to submit a revised version of the manuscript that addresses the points raised during the review process.

Please submit your revised manuscript by Jun 04 2026 11:59PM. If you will need more time than this to complete your revisions, please reply to this message or contact the journal office at plosone@plos.org. When you're ready to submit your revision, log on to https://www.editorialmanager.com/pone/ and select the 'Submissions Needing Revision' folder to locate your manuscript file.

- A letter that responds to each point raised by the academic editor and reviewer(s). You should upload this letter as a separate file labeled 'Response to Reviewers'.
- A marked-up copy of your manuscript that highlights changes made to the original version. You should upload this as a separate file labeled 'Revised Manuscript with Track Changes'.
- An unmarked version of your revised paper without tracked changes. You should upload this as a separate file labeled 'Manuscript'.

If you would like to make changes to your financial disclosure, please include your updated statement in your cover letter. Guidelines for resubmitting your figure files are available below the reviewer comments at the end of this letter.

If applicable, we recommend that you deposit your laboratory protocols in protocols.io to enhance the reproducibility of your results. Protocols.io assigns your protocol its own identifier (DOI) so that it can be cited independently in the future. For instructions see: https://journals.plos.org/plosone/s/submission-guidelines#loc-laboratory-protocols. Additionally, PLOS ONE offers an option for publishing peer-reviewed Lab Protocol articles, which describe protocols hosted on protocols.io. Read more information on sharing protocols at https://plos.org/protocols?utm_medium=editorial-email&utm_source=authorletters&utm_campaign=protocols.

As the corresponding author, your ORCID iD is verified in the submission system and will appear in the published article. PLOS supports the use of ORCID, and we encourage all coauthors to register for an ORCID iD and use it as well. Please encourage your coauthors to verify their ORCID iD within the submission system before final acceptance, as unverified ORCID iDs will not appear in the published article. *Only*  the individual author can complete the verification step; PLOS staff *cannot*  verify ORCID iDs on behalf of authors.

We look forward to receiving your revised manuscript.

Kind regards,

Jennifer Tucker, PhD

Staff Editor

PLOS One

Journal Requirements:

When submitting your revision, we need you to address these additional requirements.

1. Please ensure that your manuscript meets PLOS ONE's style requirements, including those for file naming. The PLOS ONE style templates can be found at

https://journals.plos.org/plosone/s/file?id=wjVg/PLOSOne_formatting_sample_main_body.pdf and https://journals.plos.org/plosone/s/file?id=ba62/PLOSOne_formatting_sample_title_authors_affiliations.pdf

2. Thank you for stating the following financial disclosure:

[This research has been funded by Canadian Institutes of Health Research grant#IT7 - 188084.].

Please state what role the funders took in the study.  If the funders had no role, please state: "The funders had no role in study design, data collection and analysis, decision to publish, or preparation of the manuscript."

If this statement is not correct you must amend it as needed.

Please include this amended Role of Funder statement in your cover letter; we will change the online submission form on your behalf.

3. Please remove any funding-related text from the manuscript. Funding information should not appear in any section or other areas of your manuscript. We will only publish funding information present in the Funding Statement section of the online submission form.

4. Thank you for stating the following in the Competing Interests section:

[I have read the journal's policy and the authors of this manuscript have the following competing interests: Authors comprise a mix of dual‑role program developer/implementer–evaluators and individuals focused exclusively on evaluation. This distribution of roles mitigates potential conflicts of interest.].

Please confirm that this does not alter your adherence to all PLOS ONE policies on sharing data and materials, by including the following statement: "This does not alter our adherence to PLOS ONE policies on sharing data and materials.” (as detailed online in our guide for authors http://journals.plos.org/plosone/s/competing-interests).  If there are restrictions on sharing of data and/or materials, please state these. Please note that we cannot proceed with consideration of your article until this information has been declared.

Please include your updated Competing Interests statement in your cover letter; we will change the online submission form on your behalf.

5. Please amend your list of authors on the manuscript to ensure that each author is linked to an affiliation. Authors’ affiliations should reflect the institution where the work was done (if authors moved subsequently, you can also list the new affiliation stating “current affiliation:….” as necessary).

6. Your ethics statement should only appear in the Methods section of your manuscript. If your ethics statement is written in any section besides the Methods, please move it to the Methods section and delete it from any other section. Please ensure that your ethics statement is included in your manuscript, as the ethics statement entered into the online submission form will not be published alongside your manuscript.

7. We note you have included a table to which you do not refer in the text of your manuscript. Please ensure that you refer to Tables 1-2 in your text; if accepted, production will need this reference to link the reader to the Tables.

8. Please include captions for your Supporting Information files at the end of your manuscript, and update any in-text citations to match accordingly. Please see our Supporting Information guidelines for more information: http://journals.plos.org/plosone/s/supporting-information.

If the reviewer comments include a recommendation to cite specific previously published works, please review and evaluate these publications to determine whether they are relevant and should be cited. There is no requirement to cite these works unless the editor has indicated otherwise.

Please review your reference list to ensure that it is complete and correct. If you have cited papers that have been retracted, please include the rationale for doing so in the manuscript text, or remove these references and replace them with relevant current references. Any changes to the reference list should be mentioned in the rebuttal letter that accompanies your revised manuscript. If you need to cite a retracted article, indicate the article’s retracted status in the References list and also include a citation and full reference for the retraction notice.

[Note: HTML markup is below. Please do not edit.]

Reviewers' comments:

Reviewer's Responses to Questions

**Comments to the Author**

1. Does the manuscript provide a valid rationale for the proposed study, with clearly identified and justified research questions?

Reviewer #1: Yes

**********

2. Is the protocol technically sound and planned in a manner that will lead to a meaningful outcome and allow testing the stated hypotheses?

Reviewer #1: Yes

**********

3. Is the methodology feasible and described in sufficient detail to allow the work to be replicable?

Reviewer #1: No

**********

4. Have the authors described where all data underlying the findings will be made available when the study is complete?

The PLOS Data policy

Reviewer #1: Yes

**********

5. Is the manuscript presented in an intelligible fashion and written in standard English?

Reviewer #1: Yes

**********

Please use the space provided to explain your answers to the questions above and, if applicable, provide comments about issues authors must address before this protocol can be accepted for publication. You may also include additional comments for the author, including concerns about research or publication ethics.

You may also provide optional suggestions and comments to authors that they might find helpful in planning their study.

Reviewer #1: Congratulations on the article and on the research you propose to carry out. Integrated healthcare in COPD is crucial to improving, for example, adherence and the long-term effects of interventions such as pulmonary rehabilitation and self-management programs. It will be very interesting to understand how you plan to implement these clinical pathways in this setting and what impact they might have locally. I believe this article is well-written, scientifically relevant, and should be published, with some revisions:

Introduction

- Line 59-60 - Could you present some data on estimates of undiagnosed patients?

- Line 63 - How is pharmacological care inappropriate or poorly aligned? Some practical examples could be useful.

- Line 80 - It could be interesting to mention the lack of clear guidelines on how to implement these self-management interventions.

- Line 126-128 - Were stakeholders involved in the development of this clinical pathway? It would be a good opportunity to frame, with evidence, the importance of developing health interventions and solutions that involve stakeholders, in order to improve adherence and effectiveness.

Methods:

- Are there any reporting guidelines you could use to guide the study’s development and reporting?

- Line 192-194 - How is the coordination made between different health isntituitions? Was there an improvement in digital services, for example, was interoperability accounted for?

- Line 229 - What does data collection consist of at this stage? Interviews? Who are the stakeholders included? Yous should present this information in a more comprehensive manner.

- Line 266-267 - Is there an evidence-based justification for this sample size decision?

Conclusion

- The first paragraph could describe more comprehensively and clearly the clinical and future research implications of this study.

Line 348-349 - Are there any barriers to implementing the protocol? Is there a mitigation plan for those risks?

Typos:

- Line 90 "throughput"

- Line 162 - "shap[e] the mechanisms through which [an]"

- Line 224 - "leraning"

**********

what does this mean?). If published, this will include your full peer review and any attached files.

If you choose “no”, your identity will remain anonymous but your review may still be made public.

**Do you want your identity to be public for this peer review?**  For information about this choice, including consent withdrawal, please see our Privacy Policy

Reviewer #1: No

**********

[NOTE: If reviewer comments were submitted as an attachment file, they will be attached to this email and accessible via the submission site. Please log into your account, locate the manuscript record, and check for the action link "View Attachments". If this link does not appear, there are no attachment files.]

To ensure your figures meet our technical requirements, please review our figure guidelines: https://journals.plos.org/plosone/s/figures

You may also use PLOS’s free figure tool, NAAS, to help you prepare publication quality figures: https://journals.plos.org/plosone/s/figures#loc-tools-for-figure-preparation.

NAAS will assess whether your figures meet our technical requirements by comparing each figure against our figure specifications.

# Author comment: Author response to Decision Letter 1

**DOI:** 10.1371/journal.pone.0359299.r002

15 Jun 2026

June 11th 2026

We thank the editor and reviewer for their review of the paper and notes for improvement. We have addressed the feedback below, with our responses highlighted in yellow [in the attached file] and references to the changes in the manuscript (line numbers) included in parenthesis.

We have also appended a change to authorship form that outlines our requests for change and their justifications.

Kathrina Mazurik

EDITOR FEEDBACK

1. Please ensure that your manuscript meets PLOS ONE's style requirements, including those for file naming. The PLOS ONE style templates can be found at

https://journals.plos.org/plosone/s/file?id=wjVg/PLOSOne_formatting_sample_main_body.pdf and https://journals.plos.org/plosone/s/file?id=ba62/PLOSOne_formatting_sample_title_authors_affiliations.pdf

We have aligned our formatting with the above guidelines (see tracked changes throughout document).

2. Thank you for stating the following financial disclosure:

[This research has been funded by Canadian Institutes of Health Research grant#IT7 - 188084.].

Please state what role the funders took in the study. If the funders had no role, please state: "The funders had no role in study design, data collection and analysis, decision to publish, or preparation of the manuscript."

If this statement is not correct you must amend it as needed.

Please include this amended Role of Funder statement in your cover letter; we will change the online submission form on your behalf.

Please revise the Role of Funder statement to the following : “This research has been funded by Canadian Institutes of Health Research grant #IT7 -188084. The funders had no role in study design, data collection and analysis, decision to publish, or preparation of the manuscript.”

3. Please remove any funding-related text from the manuscript. Funding information should not appear in any section or other areas of your manuscript. We will only publish funding information present in the Funding Statement section of the online submission form.

We have removed this information.

4. Thank you for stating the following in the Competing Interests section:

[I have read the journal's policy and the authors of this manuscript have the following competing interests: Authors comprise a mix of dual role program developer/implementer–evaluators and individuals focused exclusively on evaluation. This distribution of roles mitigates potential conflicts of interest.].

Please confirm that this does not alter your adherence to all PLOS ONE policies on sharing data and materials, by including the following statement: "This does not alter our adherence to PLOS ONE policies on sharing data and materials.” (as detailed online in our guide for authors http://journals.plos.org/plosone/s/competing-interests). If there are restrictions on sharing of data and/or materials, please state these. Please note that we cannot proceed with consideration of your article until this information has been declared.

Please include your updated Competing Interests statement in your cover letter; we will change the online submission form on your behalf.

Please revise the Competing Interests statement to the following: “I have read the journal's policy and the authors of this manuscript have the following competing interests: Authors comprise a mix of dual role program developer/implementer–evaluators and individuals focused exclusively on evaluation. This distribution of roles mitigates potential conflicts of interest. This does not alter our adherence to PLOS ONE policies on sharing data and materials.”

5. Please amend your list of authors on the manuscript to ensure that each author is linked to an affiliation. Authors’ affiliations should reflect the institution where the work was done (if authors moved subsequently, you can also list the new affiliation stating “current affiliation:….” as necessary).

The Patient Partners on our team do not have a formal affiliation to any institution (e.g., University of Saskatchewan or Saskatchewan Health Authority). Therefore, we have revised the list of authors to link these patient partners to their towns/cities of residence to highlight geographic representation (lines #14-17).

6. Your ethics statement should only appear in the Methods section of your manuscript. If your ethics statement is written in any section besides the Methods, please move it to the Methods section and delete it from any other section. Please ensure that your ethics statement is included in your manuscript, as the ethics statement entered into the online submission form will not be published alongside your manuscript.

We have removed the additional, unnecessary Ethics & Dissemination section and transferred to the Methods section all content that was not already captured in Methods (i.e., line #324).

7. We note you have included a table to which you do not refer in the text of your manuscript. Please ensure that you refer to Tables 1-2 in your text; if accepted, production will need this reference to link the reader to the Tables.

We have added references to the tables in the body of the manuscript (lines #247 and #288).

8. Please include captions for your Supporting Information files at the end of your manuscript, and update any in-text citations to match accordingly. Please see our Supporting Information guidelines for more information: http://journals.plos.org/plosone/s/supporting-information.

Our figures (2) and tables (2) are intended to be included in the body of the script. We have no supporting information files to share.

REVIEWER FEEDBACK

[Note: HTML markup is below. Please do not edit.]

Reviewers' comments:

Reviewer's Responses to Questions

Comments to the Author

1. Does the manuscript provide a valid rationale for the proposed study, with clearly identified and justified research questions?

The research question outlined is expected to address a valid academic problem or topic and contribute to the base of knowledge in the field.

Reviewer #1: Yes

________________________________________

2. Is the protocol technically sound and planned in a manner that will lead to a meaningful outcome and allow testing the stated hypotheses?

The manuscript should describe the methods in sufficient detail to prevent undisclosed flexibility in the experimental procedure or analysis pipeline, including sufficient outcome-neutral conditions (e.g. necessary controls, absence of floor or ceiling effects) to test the proposed hypotheses and a statistical power analysis where applicable. As there may be aspects of the methodology and analysis which can only be refined once the work is undertaken, authors should outline potential assumptions and explicitly describe what aspects of the proposed analyses, if any, are exploratory.

Reviewer #1: Yes

________________________________________

3. Is the methodology feasible and described in sufficient detail to allow the work to be replicable?

Descriptions of methods and materials in the protocol should be reported in sufficient detail for another researcher to reproduce all experiments and analyses. The protocol should describe the appropriate controls, sample size calculations, and replication needed to ensure that the data are robust and reproducible.

Reviewer #1: No

We will address this note with both 1) a response regarding our purpose for publishing a protocol and 2) some revisions that (though unrequested by the reviewer) support these objectives more fully.

The purpose of publishing this protocol is not to impose a rigid or positivist structure on the study, but rather to promote transparency, methodological clarity, and accountability to community partners, participants, and readers. In the context of partnership-based and patient-oriented research, transparently sharing protocols helps ensure relational accountability by clearly documenting the study’s commitments to collaboration, organization-to-organization respect, and patient engagement from the outset. While realist research is inherently iterative and evolves throughout the research process, this protocol outlines the guiding methodological framework and engagement-related commitments of the study, while recognizing that method will respond to the emerging theoretical insights throughout the analytic process. In addition, we anticipated the final research manuscript may exceed the word limits of many journals. Therefore, referring to the protocol serves a practical purpose to ensure that our methods are described in detail and available to all readers.

We have taken into consideration whether there is an opportunity to improve our transparency of analysis. As a result, we have added detail to our plan for statistical analysis, including realist assumptions that must be attended to while conducting this analysis, which otherwise might be violated (lines #345-352). In addition, we have added details to the analysis of qualitative data and the steps through which inductive, deductive, and retroductive reasoning will be applied (lines #355-377).

________________________________________

4. Have the authors described where all data underlying the findings will be made available when the study is complete?

The PLOS Data policy requires authors to make all data underlying the findings described in their manuscript fully available without restriction, with rare exception, at the time of publication. The data should be provided as part of the manuscript or its supporting information, or deposited to a public repository. For example, in addition to summary statistics, the data points behind means, medians and variance measures should be available. If there are restrictions on publicly sharing data—e.g. participant privacy or use of data from a third party—those must be specified.

Reviewer #1: Yes

________________________________________

5. Is the manuscript presented in an intelligible fashion and written in standard English?

PLOS ONE does not copyedit accepted manuscripts, so the language in submitted articles must be clear, correct, and unambiguous. Any typographical or grammatical errors should be corrected at revision, so please note any specific errors here.

Reviewer #1: Yes

________________________________________

6. Review Comments to the Author

Please use the space provided to explain your answers to the questions above and, if applicable, provide comments about issues authors must address before this protocol can be accepted for publication. You may also include additional comments for the author, including concerns about research or publication ethics.

You may also provide optional suggestions and comments to authors that they might find helpful in planning their study.

(Please upload your review as an attachment if it exceeds 20,000 characters)

Reviewer #1: Congratulations on the article and on the research you propose to carry out. Integrated healthcare in COPD is crucial to improving, for example, adherence and the long-term effects of interventions such as pulmonary rehabilitation and self-management programs. It will be very interesting to understand how you plan to implement these clinical pathways in this setting and what impact they might have locally. I believe this article is well-written, scientifically relevant, and should be published, with some revisions:

Introduction

- Line 59-60 - Could you present some data on estimates of undiagnosed patients?

We have added a Canada-relevant range drawn from a review article (lines #64-65).

- Line 63 - How is pharmacological care inappropriate or poorly aligned? Some practical examples could be useful.

We have added some specific findings from a relevant Canadian study on this topic (lines #67-73).

- Line 80 - It could be interesting to mention the lack of clear guidelines on how to implement these self-management interventions.

We have added a comment to this effect and cited two relevant studies that speak to the variability of self-management interventions and the variability of patients’ self-management profiles (lines #91-95).

- Line 126-128 - Were stakeholders involved in the development of this clinical pathway? It would be a good opportunity to frame, with evidence, the importance of developing health interventions and solutions that involve stakeholders, in order to improve adherence and effectiveness.

We have indicated the various collaborations that were part of developing the pathway and also highlighted the value of this stakeholder engagement by drawing on two review studies (lines #214-219).

Methods:

- Are there any reporting guidelines you could use to guide the study’s development and reporting?

We have added a statement at the outset of the Methods regarding our adherence to RAMESES reporting standards (lines #231-232).

- Line 192-194 - How is the coordination made between different health isntituitions? Was there an improvement in digital services, for example, was interoperability accounted for?

We have clarified that the improvements in coordination are largely due to increased capacity and trusted interpretation with centralized referrals for spirometry, spirometry test results being readily available over electronic health record viewers, enhanced transition between acute and community care, and hospital discharge bundles (lines #207-211).

- Line 229 - What does data collection consist of at this stage? Interviews? Who are the stakeholders included? Yous should present this information in a more comprehensive manner.

In Phase 1, there is no data collection as in realist evaluation the purpose is to initially form rough program theories. We have added a global statement to reinforce this (lines #255-256), used clearer language in Table 2 (so that it is consistent with what is in the text), clarified that the stakeholders who are part of the consultation are within the Implementation Science Team (line #282) and moved the table earlier in the text so that it more clearly sign-posts the following write-up.

- Line 266-267 - Is there an evidence-based justification for this sample size decision?

We have provided a justification that aligns with realist understandings of sampling (lines #298-301).

Conclusion

- The first paragraph could describe more comprehensively and clearly the clinical and future research implications of this study.

We have added further details to highlight the potential research implications of this study as well noting that these pathways also may serve primary care practitioners (lines #388-398).

Line 348-349 - Are there any barriers to implementing the protocol? Is there a mitigation plan for those risks?

We do not foresee any barriers implementing the protocol.

Typos:

- Line 90 "throughput"

This was intentional spelling, meant to characterize the number of patients that can be treated within the system (as opposed to “throughout” – as in care “throughout” the system).

- Line 162 - "shap[e] the mechanisms through which [an]"

We have included the square brackets to indicate that the quotation has been adapted grammatically.

- Line 224 - "leraning"

We have corrected the error.

________________________________________

7. PLOS authors have the option to publish the peer review history of their article (what does this mean?). If published, this will include your full peer review and any attached files.

If you choose “no”, your identity will remain anonymous but your review may still be made public.

Do you want your identity to be public for this peer review? For information about this choice, including consent withdrawal, please see our Privacy Policy.

Reviewer #1: No

Additional minor changes:

In addition to the above, we have included minor revisions:

- We clarified that the 43% of patients who didn’t receive smoking cessation support was among current smokers with COPD (as opposed to the denominator reflecting the entire group, including non-smokers) (line #88)

- We added precision to our wording concerning the relationship between evidence and theory (line #162)

- We clarified that program theories will also be presented to patient family partners who are part of the implementation science team (not only pathway leads)

- We corrected an address for one of the authors and a middle initial for one of the authors

- We corrected a duplicate citation of Ban et al. (2012) (formerly reference # 25 and #32)

Attachment Submitted filename: Responses to editor and reviewer June 11 2026.docx

# Peer review: Decision Letter 1

**DOI:** 10.1371/journal.pone.0359299.r003

**Authors:** Vipula Bataduwaarachchi

3 Sep 2026

Dear Dr.  Mazurik,

Thank you for submitting your manuscript to PLOS One. After careful consideration, we feel that it has merit but does not fully meet PLOS One’s publication criteria as it currently stands. Therefore, we invite you to submit a revised version of the manuscript that addresses the points raised during the review process.

Please submit your revised manuscript by Oct 18 2026 11:59PM. If you will need more time than this to complete your revisions, please reply to this message or contact the journal office at plosone@plos.org. When you're ready to submit your revision, log on to https://www.editorialmanager.com/pone/ and select the 'Submissions Needing Revision' folder to locate your manuscript file.

- A letter that responds to each point raised by the academic editor and reviewer(s). You should upload this letter as a separate file labelled 'Response to Reviewers'.
- A marked-up copy of your manuscript that highlights changes made to the original version. You should upload this as a separate file labelled 'Revised Manuscript with Track Changes'.
- An unmarked version of your revised paper without tracked changes. You should upload this as a separate file labelled 'Manuscript'.

If you would like to make changes to your financial disclosure, please include your updated statement in your cover letter. Guidelines for resubmitting your figure files are available below the reviewer comments at the end of this letter.

If applicable, we recommend that you deposit your laboratory protocols in protocols.io to enhance the reproducibility of your results. Protocols.io assigns your protocol its own identifier (DOI) so that it can be cited independently in the future. For instructions see: https://journals.plos.org/plosone/s/submission-guidelines#loc-laboratory-protocols. Additionally, PLOS One offers an option for publishing peer-reviewed Lab Protocol articles, which describe protocols hosted on protocols.io. Read more information on sharing protocols at https://plos.org/protocols?utm_medium=editorial-email&utm_source=authorletters&utm_campaign=protocols.

As the corresponding author, your ORCID iD is verified in the submission system and will appear in the published article. PLOS supports the use of ORCID, and we encourage all coauthors to register for an ORCID iD and use it as well. Please encourage your coauthors to verify their ORCID iD within the submission system before final acceptance, as unverified ORCID iDs will not appear in the published article. *Only*  the individual author can complete the verification step; PLOS staff *cannot*  verify ORCID iDs on behalf of authors.

We look forward to receiving your revised manuscript.

Kind regards,

Vipula Rasanga Bataduwaarachchi, MD

Academic Editor

PLOS One

Journal Requirements:

If the reviewer comments include a recommendation to cite specific previously published works, please review and evaluate these publications to determine whether they are relevant and should be cited. There is no requirement to cite these works unless the editor has indicated otherwise.

Please review your reference list to ensure that it is complete and correct. If you have cited papers that have been retracted, please include the rationale for doing so in the manuscript text, or remove these references and replace them with relevant current references. Any changes to the reference list should be mentioned in the rebuttal letter that accompanies your revised manuscript. If you need to cite a retracted article, indicate the article’s retracted status in the References list and also include a citation and full reference for the retraction notice.

Additional Editor Comments:

This study protocol outlines a realist evaluation of an integrated clinical pathway for chronic obstructive pulmonary disease (COPD) implemented in Saskatchewan, Canada. This is a timely and relevant protocol that addresses a high-burden condition and uses an evaluation approach well suited to complex, system-level interventions.

Please check these

1. In the tracked-changes manuscript, Candace Skrapek is included in the author list (Line 18), but she is omitted from both the clean manuscript author list and the "Order of Authors" metadata on Editorial Manager. Clarify whether she meets ICMJE criteria for authorship or if her contribution belongs solely under Acknowledgements (where she is currently thanked in Line 449).

2. Affiliation numbers in the tracked-changes manuscript for Maggie King (5), Kim Bell (5), Brenda Andreas (5), and Candace Skrapek (5) clash with the numbered institutional keys (where key 5 is listed as Maple Creek, 6 as North Battleford, and 7 as Regina). Ensure numeric superscript affiliations match the corresponding city/institution entries across all versions.

3. In the clean manuscript text, Figure 1 (Line 193) and Figure 2 (Line 249) are embedded via placeholder text, Hope you will be providing original figures separately.

4. Table 2 is formatted twice in the tracked-changes version (once in a clear block and once scattered across lines 345–351 due to line-break artefacts during typesetting). Ensure the stray text from the duplicate table in the revision draft is completely removed.

5. Line 46: "Findings will be shared through integrated knowledge transition..." — The correct, standard implementation science terminology is Integrated Knowledge Translation (IKT) rather than "knowledge transition."

6. Line 207: "...emphasizes early identification with available spirometry with trusted spirometry..." — Contains accidental repetitive wording. Rephrase to "emphasizes early identification with available, trusted spirometry..."

7. Line 310: "...a minimum of one participant and a maximum of ten participants to represent each context of interest." — Typo in double spacing between "of" and "ten".

8. To strengthen the body of the manuscript (Lines 345–377), consider adding a brief statement on data/theoretical saturation within realist sampling logic (i.e., recruitment within each context continues until conceptual depth and theory-testing capacity are achieved).

9. In the Methods section (Lines 330–338), specify the exact date range for historical baseline data (e.g., September 2017 to present) to clarify how pre-pathway baseline outcomes will be compared against post-implementation trends in Saskatchewan.

10. Verify that the RAMESES II checklist is uploaded as a Supporting Information file (S1 File) and explicitly referenced in the manuscript text under Methods, as required by PLOS ONE for realist study designs.

[Note: HTML markup is below. Please do not edit.]

Reviewers' comments:

Reviewer's Responses to Questions

**Comments to the Author**

1. Does the manuscript provide a valid rationale for the proposed study, with clearly identified and justified research questions?

Reviewer #1: Yes

**********

2. Is the protocol technically sound and planned in a manner that will lead to a meaningful outcome and allow testing the stated hypotheses?

Reviewer #1: Yes

**********

3. Is the methodology feasible and described in sufficient detail to allow the work to be replicable?

Reviewer #1: Yes

**********

4. Have the authors described where all data underlying the findings will be made available when the study is complete?

The PLOS Data policy

Reviewer #1: Yes

**********

5. Is the manuscript presented in an intelligible fashion and written in standard English?

Reviewer #1: Yes

**********

Please use the space provided to explain your answers to the questions above and, if applicable, provide comments about issues authors must address before this protocol can be accepted for publication. You may also include additional comments for the author, including concerns about research or publication ethics.

You may also provide optional suggestions and comments to authors that they might find helpful in planning their study.

Reviewer #1: I want to appreciate the attention to detail and the effort from the authors to answer the review and address the comments and suggestions made.

Regarding the question about “Descriptions of methods and materials in the protocol should be reported in sufficient detail for another researcher to reproduce all experiments and analyses”, it was more related to the reproducibility of methods. I understand the relevance and importance of publishing this study protocol and I appreciate the effort to improve the transparency of the methodology, particularly the data analysis section.

I just recommend a last review of text and spacing throughout the manuscript (noticed a space missing in line 73).

I consider this a relevant study protocol, and it should be published. I’m eager to see its results. I wish the authors success in this project.

**********

what does this mean?). If published, this will include your full peer review and any attached files.

If you choose “no”, your identity will remain anonymous but your review may still be made public.

**Do you want your identity to be public for this peer review?**  For information about this choice, including consent withdrawal, please see our Privacy Policy

Reviewer #1: No

**********

[NOTE: If reviewer comments were submitted as an attachment file, they will be attached to this email and accessible via the submission site. Please log into your account, locate the manuscript record, and check for the action link "View Attachments". If this link does not appear, there are no attachment files.]

To ensure your figures meet our technical requirements, please review our figure guidelines: https://journals.plos.org/plosone/s/figures

You may also use PLOS’s free figure tool, NAAS, to help you prepare publication quality figures: https://journals.plos.org/plosone/s/figures#loc-tools-for-figure-preparation.

NAAS will assess whether your figures meet our technical requirements by comparing each figure against our figure specifications.

# Author comment: Author response to Decision Letter 2

**DOI:** 10.1371/journal.pone.0359299.r004

10 Sep 2026

September 10th 2026

*Below are our responses to the editor:

Please check these

1. In the tracked-changes manuscript, Candace Skrapek is included in the author list (Line 18), but she is omitted from both the clean manuscript author list and the "Order of Authors" metadata on Editorial Manager. Clarify whether she meets ICMJE criteria for authorship or if her contribution belongs solely under Acknowledgements (where she is currently thanked in Line 449).

*Skrapek has been removed as an author and is listed under Acknowledgments instead. This is reflected in the tracked changes document – Sept 10 version as well as the clean copy.

2. Affiliation numbers in the tracked-changes manuscript for Maggie King (5), Kim Bell (5), Brenda Andreas (5), and Candace Skrapek (5) clash with the numbered institutional keys (where key 5 is listed as Maple Creek, 6 as North Battleford, and 7 as Regina). Ensure numeric superscript affiliations match the corresponding city/institution entries across all versions.

*The affiliation numbers align (Andreas=5; King=6, Bell=7) and this is reflected in the tracked changes document – Sept 10 version and the clean copy.

3. In the clean manuscript text, Figure 1 (Line 193) and Figure 2 (Line 249) are embedded via placeholder text, Hope you will be providing original figures separately.

*We confirm that the figures have been uploaded separately.

4. Table 2 is formatted twice in the tracked-changes version (once in a clear block and once scattered across lines 345–351 due to line-break artefacts during typesetting). Ensure the stray text from the duplicate table in the revision draft is completely removed.

*This appears to be a non-issue in the clean version and when “no mark-up” setting is activated in the tracked-changes version.

5. Line 46: "Findings will be shared through integrated knowledge transition..." — The correct, standard implementation science terminology is Integrated Knowledge Translation (IKT) rather than "knowledge transition."

*We have made this correction.

6. Line 207: "...emphasizes early identification with available spirometry with trusted spirometry..." — Contains accidental repetitive wording. Rephrase to "emphasizes early identification with available, trusted spirometry..."

*We have made this change.

7. Line 310: "...a minimum of one participant and a maximum of ten participants to represent each context of interest." — Typo in double spacing between "of" and "ten".

*This has been resolved.

8. To strengthen the body of the manuscript (Lines 345–377), consider adding a brief statement on data/theoretical saturation within realist sampling logic (i.e., recruitment within each context continues until conceptual depth and theory-testing capacity are achieved).

*The concept of “theoretical saturation” draws from grounded theory methodology. The key texts we’ve drawn on to inform our global approach and approach to sampling make no mention of this concept and we prefer our current phrasing, which emphasizes the resolution of gaps and contradictions between evidence and theory. See Manzano (2016), Mukumbang et al. (2016), and Stoszkowski & Collins (2022) as examples of realist researchers who have, similarly, eschewed the concept of “saturation” in favor of criteria or processes that fit more cleanly within the realist paradigm.

9. In the Methods section (Lines 330–338), specify the exact date range for historical baseline data (e.g., September 2017 to present) to clarify how pre-pathway baseline outcomes will be compared against post-implementation trends in Saskatchewan.

*We have revised “present” to “August 2028”.

10. Verify that the RAMESES II checklist is uploaded as a Supporting Information file (S1 File) and explicitly referenced in the manuscript text under Methods, as required by PLOS ONE for realist study designs.

*We can confirm the RAMESES reporting standards are uploaded as a supplementary file, however there is no “checklist” as these are published in rubric form. The RAMESES reporting standards are referenced in the Overview of study phases within the section Methods and analysis.

[Note: HTML markup is below. Please do not edit.]

Reviewers' comments:

Reviewer's Responses to Questions

Comments to the Author

1. Does the manuscript provide a valid rationale for the proposed study, with clearly identified and justified research questions?

The research question outlined is expected to address a valid academic problem or topic and contribute to the base of knowledge in the field.

Reviewer #1: Yes

________________________________________

2. Is the protocol technically sound and planned in a manner that will lead to a meaningful outcome and allow testing the stated hypotheses?

The manuscript should describe the methods in sufficient detail to prevent undisclosed flexibility in the experimental procedure or analysis pipeline, including sufficient outcome-neutral conditions (e.g. necessary controls, absence of floor or ceiling effects) to test the proposed hypotheses and a statistical power analysis where applicable. As there may be aspects of the methodology and analysis which can only be refined once the work is undertaken, authors should outline potential assumptions and explicitly describe what aspects of the proposed analyses, if any, are exploratory.

Reviewer #1: Yes

________________________________________

3. Is the methodology feasible and described in sufficient detail to allow the work to be replicable?

Descriptions of methods and materials in the protocol should be reported in sufficient detail for another researcher to reproduce all experiments and analyses. The protocol should describe the appropriate controls, sample size calculations, and replication needed to ensure that the data are robust and reproducible.

Reviewer #1: Yes

________________________________________

4. Have the authors described where all data underlying the findings will be made available when the study is complete?

The PLOS Data policy requires authors to make all data underlying the findings described in their manuscript fully available without restriction, with rare exception, at the time of publication. The data should be provided as part of the manuscript or its supporting information, or deposited to a public repository. For example, in addition to summary statistics, the data points behind means, medians and variance measures should be available. If there are restrictions on publicly sharing data—e.g. participant privacy or use of data from a third party—those must be specified.

Reviewer #1: Yes

________________________________________

5. Is the manuscript presented in an intelligible fashion and written in standard English?

PLOS ONE does not copyedit accepted manuscripts, so the language in submitted articles must be clear, correct, and unambiguous. Any typographical or grammatical errors should be corrected at revision, so please note any specific errors here.

Reviewer #1: Yes

________________________________________

6. Review Comments to the Author

Please use the space provided to explain your answers to the questions above and, if applicable, provide comments about issues authors must address before this protocol can be accepted for publication. You may also include additional comments for the author, including concerns about research or publication ethics.

You may also provide optional suggestions and comments to authors that they might find helpful in planning their study.

(Please upload your review as an attachment if it exceeds 20,000 characters)

Reviewer #1: I want to appreciate the attention to detail and the effort from the authors to answer the review and address the comments and suggestions made.

Regarding the question about “Descriptions of methods and materials in the protocol should be reported in sufficient detail for another researcher to reproduce all experiments and analyses”, it was more related to the reproducibility of methods. I understand the relevance and importance of publishing this study protocol and I appreciate the effort to improve the transparency of the methodology, particularly the data analysis section.

I just recommend a last review of text and spacing throughout the manuscript (noticed a space missing in line 73).

I consider this a relevant study protocol, and it should be published. I’m eager to see its results. I wish the authors success in this project.

*We thank the reviewer for the comments.

We have added a space on line 73 between “symptomatic” and the in-text citation.

Attachment Submitted filename: Responses to editor & reviewer.docx

# Editor report: Decision Letter 2

**DOI:** 10.1371/journal.pone.0359299.r005

**Authors:** Vipula Bataduwaarachchi

13 Sep 2026

Protocol for a Realist Evaluation of a COPD Clinical Pathway in Saskatchewan, Canada

PONE-D-26-05808R2

Dear Dr. Mazurik,

We’re pleased to inform you that your manuscript has been judged scientifically suitable for publication and will be formally accepted for publication once it meets all outstanding technical requirements.

Within one week, you’ll receive an e-mail detailing the required amendments. When these have been addressed, you’ll receive a formal acceptance letter and your manuscript will be scheduled for publication.

An invoice will be generated when your article is formally accepted. Please note, if your institution has a publishing partnership with PLOS and your article meets the relevant criteria, all or part of your publication costs will be covered. Please make sure your user information is up-to-date by logging into Editorial Manager at Editorial Manager® and clicking the ‘Update My Information' link at the top of the page. For questions related to billing, please contact billing support.

If your institution or institutions have a press office, please notify them about your upcoming paper to help maximize its impact. If they’ll be preparing press materials, please inform our press team as soon as possible -- no later than 48 hours after receiving the formal acceptance. Your manuscript will remain under strict press embargo until 2 pm Eastern Time on the date of publication. For more information, please contact onepress@plos.org.

Kind regards,

Vipula Rasanga Bataduwaarachchi, MD

Academic Editor

PLOS One

# Editor report: Acceptance letter

**DOI:** 10.1371/journal.pone.0359299.r006

**Authors:** Vipula Bataduwaarachchi

PONE-D-26-05808R2

PLOS One

Dear Dr. Mazurik,

I'm pleased to inform you that your manuscript has been deemed suitable for publication in PLOS One. Congratulations! Your manuscript is now being handed over to our production team.

At this stage, our production department will prepare your paper for publication. This includes ensuring the following:

* All references, tables, and figures are properly cited

* All relevant supporting information is included in the manuscript submission,

* There are no issues that prevent the paper from being properly typeset

You will receive further instructions from the production team, including instructions on how to review your proof when it is ready. Please keep in mind that we are working through a large volume of accepted articles, so please give us a few days to review your paper and let you know the next and final steps.

Lastly, if your institution or institutions have a press office, please let them know about your upcoming paper now to help maximize its impact. If they'll be preparing press materials, please inform our press team within the next 48 hours. Your manuscript will remain under strict press embargo until 2 pm Eastern Time on the date of publication. For more information, please contact onepress@plos.org.

You will receive an invoice from PLOS for your publication fee after your manuscript has reached the completed accept phase. If you receive an email requesting payment before acceptance or for any other service, this may be a phishing scheme. Learn how to identify phishing emails and protect your accounts at https://explore.plos.org/phishing.

If we can help with anything else, please email us at customercare@plos.org.

Thank you for submitting your work to PLOS One and supporting open access.

Kind regards,

PLOS One Editorial Office Staff

on behalf of

Dr. Vipula Rasanga Bataduwaarachchi

Academic Editor

PLOS One
